# Part II: superconductivity observed in magnetically separated nanoscale anatase titania at ambient temperature and pressure in an aqueous environment at its point of zero charge

**DOI:** 10.1039/d4ra01165a

**Published:** 2024-09-24

**Authors:** Miriam Leffler, Jared Fee, Seth March, Yang Wu, Steven L. Suib

**Affiliations:** a Department of Chemistry, University of Connecticut USA Miriamleffler1066@gmail.com jare.fee@uconn.edu seth.march@uconn.edu yang.wu@uconn.edu steven.suib@uconn.edu

## Abstract

This is the first work to investigate if and/or how changes in the surface structure/properties affect the charge transfer resistance (*R*_CT_) of anatase titania with decreasing particle size. It was accomplished by measuring the *R*_CT_ (Ω) of same weight anatase titania pellets, with particle sizes ranging from 5.31 nm to 142.61 nm. Measurements were made using Electrochemical Impedance Spectroscopy (EIS) at each material's point of zero charge (PZC). Results demonstrated two regions of *R*_CT_. Above an average primary particle diameter of 23.54 nm, *R*_CT_ remained essentially constant. Below, this diameter the *R*_CT_ value first increased significantly, then decreased almost linearly toward zero. The projected average primary particle diameter where the materials *R*_CT_ was projected to reach zero resistance is at a diameter of approximately 4.39 nm. A simple test was then developed to determine if at a small enough particle size the material would be affected by an external magnetic field. It was found that a sample with an average particle diameter of 12.689 nm, formed fine needles/threads of particles in deionized water, perpendicular to the settled powder at the base of the potash tube. This led to the development of a simple magnetic separation method to obtain strongly diamagnetic material from a parent population with an average primary particle diameter of 5.31 nm. A pellet consisting of these magnetically separated particles was then pressed at the same weight and pressure as the prior samples. The pellet's *R*_CT_ was then measured using EIS under the identical conditions as the prior samples. EIS results of the magnetically separated particles in pellet form, under multiple conditions, resulted in Nyquist plots indicating the material exhibited no detectable *R*_CT_ (*i.e.*, superconductivity). Correlation of the shift in the materials *R*_CT_ with known structure/property changes for each sample with decreasing particle size allowed the development of a model explaining: (1) the significant increase in diamagnetic strength of the magnetically separated particles and (2) the mechanism controlling the material's *R*_CT_.

## Abstract 2

This is the first work to investigate if and/or how changes in the surface structure/properties affect the charge transfer resistance (*R*_CT_) of anatase titania with decreasing particle size.

## Graphical abstract

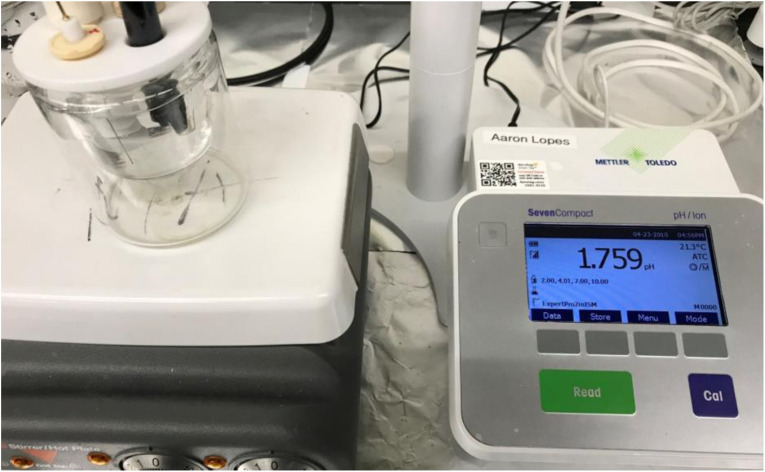

## 1 Introduction

### 1.1 Charge transfer resistance applications

Charge transfer resistance is a critical property used in multiple areas of chemistry. These include work on battery anodes,^1–3^ photosynthesis, medicine, synthesis, electronics^4^ and catalysis.^5^*R*_CT_ is also employed in determining losses in electronic component materials such as semi-conductors, capacitors, and diodes.^6–8^

Due to the extensive use of anatase titania's electronic properties^3,9–11^ it has been widely studied. In addition, critical work has been done on the particle size effect on *R*_CT_^3,12^ of this material which provided a broad range of background knowledge. This made it possible to tailor the research into the effect of changes in the materials surface structure and properties due to the particle size effect on *R*_CT_.

### 1.2 Change in charge transfer resistance with decreasing particle size

Charge transfer resistance (*R*_CT_ (Ω)) is defined as the opposition to the movement of an electron from either one atom or phase to a second atom or phase. As this resistance measurement also possesses a frequency component, it is typically referred to as impedance (active resistance). The factors affecting this value are the given amount of a specific material the current is passing through and the frequency of the current.^1^ Therefore, particle size differences in same size samples for the same phase should not affect the measured *R*_CT_, which was the finding in work by Ariyoshi, Tanimoto and Yamada.^13^ They demonstrated that significant changes in the particle size of lithium manganese oxide for same size samples exhibited little change in their *R*_CT_ values from 5 μm to 9 μm.

This though, was not the finding for work by Rai *et al.*^12^ on nanoscale anatase titania. They demonstrated that as the average primary particle diameter decreased into the nanoscale region, for same size samples, *R*_CT_ decreased almost linearly. A plot of the materials average primary particle diameter against its *R*_CT_ resulted in a linear curve. The fitted trendline gave a correlation value (*R*^2^) of 0.917 indicating the values were highly correlated and projected values of the fitted equation very reliable. The projected average primary particle diameter (*d*) for this curve, where the materials resistance would go to zero, was at *d* = 1.54 nm.

### 1.3 Effect of an electric field on the charge transfer resistance of titania

Salari *et al.*'s^3^ research on highly ordered titania nanotubes for use as supercapacitor electrodes demonstrated the effect of a voltage bias (*i.e.*, electric field) across nanoscale titania material during charge transfer resistance measurements using EIS. The average primary particle diameter for the powder population used in their work was between 30–60 nm. Nyquist plots of both their nanotubes and powder, were run with a 0.1 V bias across the samples. Their results presented Nyquist plots indicating there was no apparent *R*_CT_ (*i.e.*, bulk electrical resistance). Based on the results by Rai *et al.*'s^12^ work, though there should have been a measurable resistance in the average primary particle size used by Salari *et al.*^3^

The critical difference between these two works is that Salari *et al.*^3^ obtained the Nyquist plots with the samples under a 0.1 V bias, while Rai *et al.*'s^12^ samples were at a 0.0 V bias. Research by Rincón *et al.*^14^ on the effect of an electric field on carbon–hydrogen bonds, such as those generated by a voltage bias, determined that it lengthened the distance between the anions and cations. This creates the same effect of lengthening surface bonds with decreasing particle size, found to occur below an average diameter of approximately 28 nm in anatase titania.^15^

Based on the above information, one significant structural difference between the materials in the micron and nanoscale ranges is apparent, surface bond lengths. In the micron region, above an average diameter of ∼28 nm, surface bond lengths of anatase titania do not change. Whereas below this particle size bond lengths increase with decreasing particle size.^15^ In addition to an increase in surface bond lengths with decreasing particle size in the nanoscale region changes in other structure/properties were identified by Leffler *et al.*^16^ in anatase titania below a diameter of ≅29 nm. These include changes in the surface structure, a shift toward lower point of zero charge values, increasing bond ionic content and an increasing percent of the global surface area being positively charged. Correlation with the decrease in *R*_CT_ and changes observed by Leffler *et al.*,^16^ suggests that alterations in the surface structure/properties may be an integral part of the mechanism which controls this property.

### 1.4 Magnetic separation

#### 1.4.1 Property necessary for a magnetic separation

Based on the work of Rai *et al.*^12^ and Salari *et al.*^3^ their results suggest that at a specific particle size, under the correct conditions, anatase titania may present no discernible charge transfer resistance (*i.e.*, superconductivity). If that is the case, then the material may also exhibit either a very strong and/or super diamagnetic property.^17^ If this does occur, then it should be possible to develop a magnetic separation method to obtain particles which exhibit zero resistance.

#### 1.4.2 Dry magnetic separation

Magnetic separation methods are extensively used in mineral processing.^18^ There are two main types, dry and wet separations. The dry process involves a magnet deflecting the materials susceptible to a magnetic field along a different trajectory than the nonmagnetic material in the mix. This occurs as the feed material travels along a belt moved by rollers. At the last roller a set of magnets creates the magnetic field into which the feed enters. The magnetic field then alters the trajectory of the material magnetically susceptible as it rolls off the belt. This has the effect of creating two or three streams of material. A splitter below the belt is used to prevent the streams of material from recombining and directing the separated materials to collection bins.

A different dry magnetic separation method has also been used to segregate superconducting powders in a magnetic field by making use of the Meissner Effect.^17^ The parent powder population is cooled to below the materials transition temperature (*T*_C_) so that it is in superconducting mode. In the cooling chamber, the parent powder population sits above a strong magnet, which creates a vertical magnetic field in which the particles levitate. The more superconducting the particles, the higher they levitate in the magnetic field. Those particles which levitate at the greatest height are the most effective superconducting material and are harvested.

#### 1.4.3 Wet magnetic separation

The wet magnetic separation works in a similar manner to the dry separation method, save that segregation occurs in a liquid media. Typically, the material to be separated is dispersed in the liquid in the tank. Within the tank are permanent magnets used as the separator, deflecting the flow of magnetically susceptible material to a removal stream. The nonmagnetic material is then directed to a second refuse discharge stream. Both dry and wet magnetic separation methods are used to concentrate the desired material present in the parent population.

### 1.5 Objectives

Therefore, the objectives of this work were to:

(1) Determine the *R*_CT_ for same weight pellets with decreasing average primary particle diameters using EIS at each material's PZC. This would allow a possible projected average primary particle diameter where the material's *R*_CT_ = 0.0 Ω.

(2) Determine if a dry and/or wet magnetic separation method could be developed to concentrate anatase titania powder exhibiting either extremely strong and/or super diamagnetic characteristics from its parent population.

(3) Having obtained enough magnetically separated powder, a same weight pellet would then be pressed under the same pressure as the prior samples. The *R*_CT_ would then be measured using EIS at the materials PZC.

(4) Correlate the changes in the powder populations *R*_CT_ with the known surface structural and property changes with decreasing particle diameter to determine the possible underlying cause responsible for changes in the materials *R*_CT_ (*i.e.*, bulk electrical resistance).

Note: EIS was chosen for specific reasons.

(1) The samples would be measured under identical conditions as each material's PZC value was obtained.^16^

(2) This would allow a direct correlation between the changes between each powder population's surface properties and structure.^16^

## 2 Experimental

### 2.1 Pellet preparation

Identical amounts of anatase titania powders were measured on a Mettler scale (∼295 mg). The powder was pressed, in a 1.4 cm die, using a Brucker Powder Press at 5000 pounds per square inch (psi). After pressing each pellet was then weighed. The average weight of all seven pellets was ∼291 mg. Each pellets dimensions possessed similar diameters (∼1.4 cm) and thickness (∼1.33 mm).

### 2.2 Charge transfer resistance measurements using EIS on same weight pellets

**Fig. 1:**
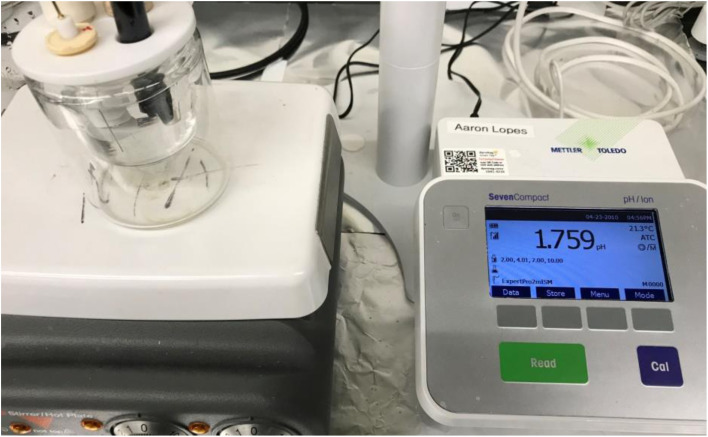
The electrical cell, clamped pellet, and pH/ion meter set-up for the EIS system used to obtain a Nyquist plot.

A Gamry Interface 1010™ Potentiostat/Galvanostat/ZRA Model 600 was used to obtain Nyquist plots for each of the seven pellets. Nyquist plots were run using Gamry Instruments Echem Analyst Software in a 3-electrode cell with a PFTE cover (Teflon). This consisted of a working electrode with a PTFE coated Pt clip, a counter electrode (Pt wire), and a reference electrode (Standard Calomel Electrode (SCE)). A SevesCompact pH/ion meter was used to monitor pH values in the 3-electrode cell. The experimental set-up is presented in Fig. 1.

Each pellet was clamped into the PTFE coated Pt clip attached to the working electrode for the run. The electrolyte pH was then adjusted to each pellet's PZC value using a 1 M or 5 M HCl or NaOH solution. The following settings and conditions were used during each run to obtain a Nyquist plot:

* DC voltage: open circuit potential (OCV)

* AC voltage: 5 mV

* Electrolyte: 0.01 M KCl

* Measured at the point of zero charge pH for each pellet: (obtained using 1 M or 5 M HCl or NaOH solution)

* Frequency range: 100 000 to 0.1 Hz

* OCV current: 0.35 A

### 2.3 Development of magnetic separation method for anatase titania

#### 2.3.1 Test for dry magnetic separation

Strong/super diamagnetic particles are known to levitate in the presence of a very strong permanent magnetic field.^17^ For this purpose, a nickel coated cylindrical (25.4 mm diameter × 25.4 mm thick), Grade N48, neodymium rare earth magnet (NdFeB) (Apex Magnets) was chosen. The magnetic field strength at the surface of the magnet was 115.2 gauss at an angle of 0°. A 0.5 g sample of anatase titania with a 12.689 nm diameter was placed directly on the Grade N48 magnet and examined for any possible effects of the magnetic field.

#### 2.3.2 Initial test to determine if a magnetic field effects anatase titania in deionized water

To determine if a magnetic field would affect the anatase titania particles in an aqueous environment, a 1 mg sample with an average primary particle diameter of 12.689 nm, was placed into a “Potash A” printed tube (Lamott). Then approximately 23 ml deionized (DI) water (ChemLab) was put into the tube and capped. The powder was then allowed to completely settle to the base of the tube. The Potash tube was then carefully placed on the same Grade N48 so as not to disturb the powder.

#### 2.3.3 Magnetic separation of anatase titania in 0.01 N HCl in the presence of a magnetic field

A simple test for anatase titania was performed using 1.0 mg anatase titania (*d* = 5.31 nm), ∼23 ml of 0.01 N HCl (LabChem) as a dispersant, a Potash tube, and the Grade N48 magnet. The titania was placed in the potash tube, the 0.01 N HCl added, then capped, and shaken vigorously. The dispersed powder was then placed on the Grade N48 magnet. The tube was exposed to ambient light for this experiment as the objective of this experiment was to detect the influence of the magnetic field on the dispersed particles. The total length of the run was 47 days.

#### 2.3.4 Particle settling in deionized water without a magnetic field present

To obtain a sample uncontaminated by a dispersant, in this case 0.01 N HCl, it was necessary to determine if anatase titania particles would settle completely without a magnetic field present. This was accomplished by placing 0.2 gm of anatase titania (*d* = 5.31 nm) into a Lamott “Potash A” tube and then adding 23 ml of DI water (ChemLab). The tube was then capped, and the tube shaken vigorously until the powder was fully dispersed in the DI water. The tube was then placed off to the side, exposed to ambient light and the level monitored each day. On day 36 a small number of particles were still diffused throughout the DI water. The rest of the particles had settled to the base of the tube.

The presence of particles in both 0.01 N HCl solution and deionized water suggested a second factor responsible for the particles remaining in solution. To determine if the presence of ambient light during settling was responsible for the particles remaining in solution a metal cup was used to cover the tube on day 53. This excluded all the ambient light from reaching the tube allowing a test of this possibility. The total run time for this experiment was 77 days.

#### 2.3.5 Magnetically separated anatase titania particles in deionized water

Having determined that ambient light results in a percentage of the particles in deionized water remaining in solution, the next step was to perform a magnetic separation with all light excluded. This was accomplished by taking the particles which had settled completely once all ambient light had been excluded, agitating the sample until they were fully dispersed and placing the tube on the cylindrical Grade N48 magnet. The tube and magnet were then covered with a cylindrical carboard box to exclude all ambient light. The total run took 15 days.

#### 2.3.6 Collection method of magnetically separated anatase titania particles in deionized water

Once the magnetic separation method was completed, the particles that had not settled, but remained in the DI water were then collected. This was accomplished by removing the particles in solution using a 200 μL Gilson pipettor and placed into a clean 50 ml polypropylene centrifuge tube. The tube was then placed on top of a warming plate (Coscori), and a glass microscope slide, partially covering the opening at the top of the tube to minimize contaminants from the atmosphere. The temperature was set to 50 °C and the DI water was allowed to evaporate until most of it was gone, thereby concentrating the sample.

The concentrated particles were then transferred from the centrifuge tube using the Gilson 200 μL pipettor to place small drops of the concentrated sample in DI water onto a clean glass microscope slide. Then the loaded slide was placed on the warming plate (*T* = 50 °C), with the top of a glass Petri dish covering it to minimize contamination from the atmosphere. Once the drops had dried, the powder was scraped off the microscope slide using the flat end of a spatulate into a glass sample jar for collection. The entire process of collecting ∼325 mg of magnetically separated anatase titania took approximately 2 years.

#### 2.3.7 Effect of neither magnetic field nor ambient light on magnetically separated anatase titania particles in deionized water

Magnetically separated particles in DI water were then covered with the metal can for approximately 60 days. This was to determine the effect of no ambient light on the magnetically separated particles still in solution with no magnetic field present.

#### 2.3.8 Magnetically separated anatase titania pellet preparation

The process described in Section 2.1 was used to press the pellet of the magnetically separated particles. The weight of this pellet was slightly higher than those formed from the commercial powders. It weighed 300.10 mg, with a diameter of 1.260 cm and a thickness of 1.30 mm. The pellet exhibited a sky-blue color, rather than the brilliant white present in the parent powder population.

#### 2.3.9 Determination of the average primary particle size for magnetically separated particles

To obtain the size and morphology of the magnetically separated particles an FEI, Talos F200X microscope utilizing a 200 kV voltage Transmission Electron Microscope (TEM) was used. Elemental mapping was unnecessary as the parent population had been confirmed to have less than 0.1% by weight of extraneous elements present in the sample.^16^ The TEM grids were prepared by combining a 2 mg sample with 20 ml of solvent (ethanol: Fischer Scientific) and sonicated for 5 minutes. The solution was then dispersed on a copper grid dropwise *via* a pipet. Multiple micrographs were taken of the magnetically separated powder population.

#### 2.3.10 Measurement of charge transfer resistance of magnetically separated particles

The method described in Section 2.2 was used to measure and press the same weight pellet of magnetically separated particles. To determine the pH_PZC_ for the magnetically separated particles, a plot of PZC values against their particles *R*_CT_ was used to extrapolate the pH_PZC_ at which *R*_CT_ = 0.0. The 0.01 M KCl electrolyte was then adjusted to the projected pH_PZC_ for the magnetically separated particles, using 1 M and 5 M HCl. Three runs were then made. The first two runs were for the sample loaded in the 3-electrode cell with a PFTE covered Pt clip (Teflon), at a voltage bias of 0.1 V, and the second at 0.0 V bias. The third run was performed with no sample in the PFTE coated Pt clip, at 0.0 bias voltage applied, to measure the unloaded system for comparison with the first two runs with the sample present.

## 3 Results

### 3.1 Changes in charge transfer resistance with decreasing particle size

#### 3.1.1 Nyquist Plots

**Fig. 2:**
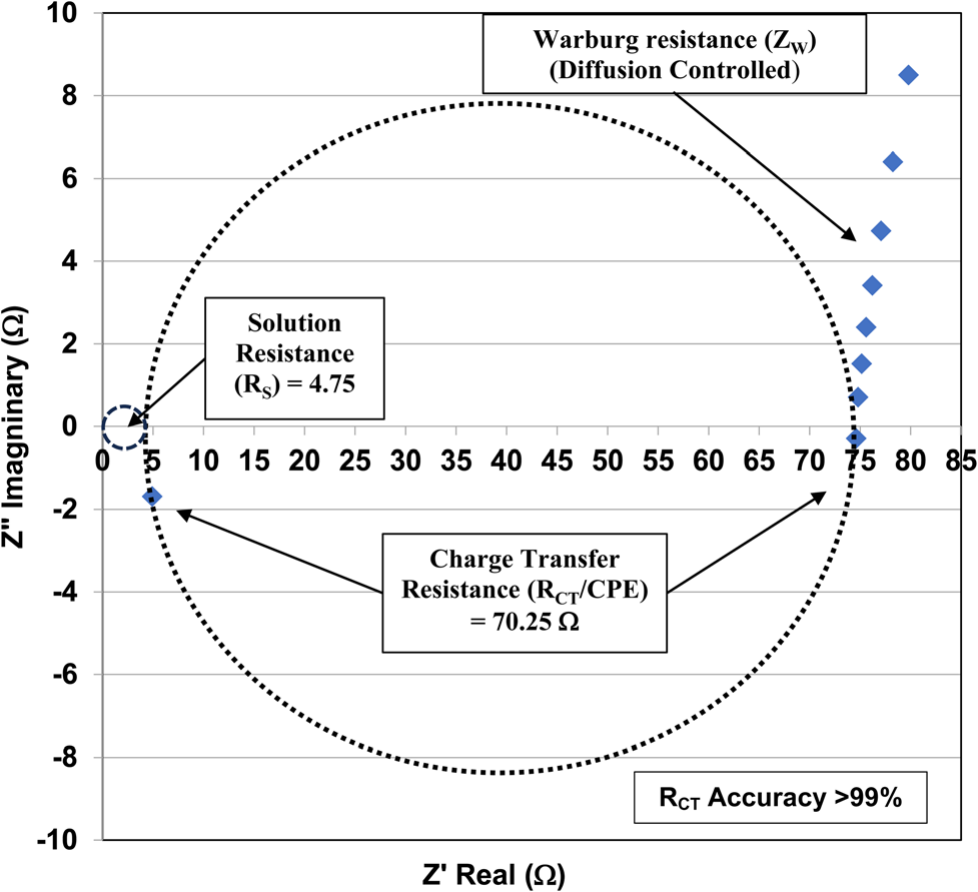
The Nyquist plot for the anatase titania pellet with an average primary particle diameter of 5.31 nm at its pH_PZC_ = 1.75.

Fig. 2 is representative of the Nyquist plots obtained for each commercial sample. The sample measured is for the powder population with an average primary particle diameter of 5.31 nm and a pH_PZC_ = 1.75. Circles were fitted over the data points to identify the semi-circles for both solution resistance (*R*_S_) and *R*_CT_ of the pellet. The data points below the real axis (*Z*′) indicate the *R*_CT_ had a negative resistance, which occurs in semi-conductors, such as anatase titania. This is due to the current decreasing during voltage increases in these types of material.

**Fig. 3:**
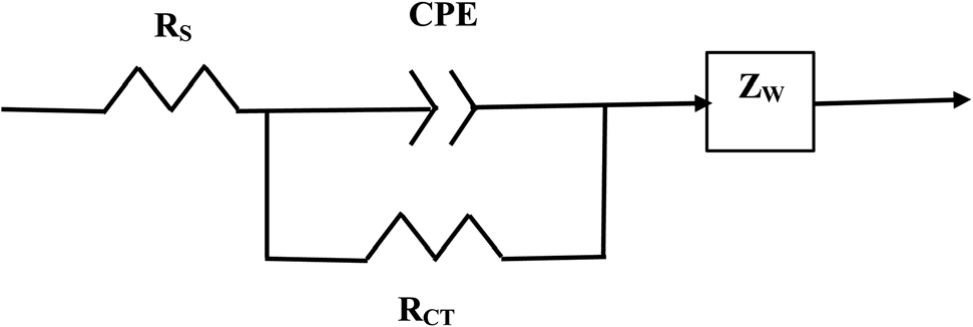
The equivalent circuit for the Nyquist Plot/system in Fig. 2.

The element in a circuit diagram typically used in parallel with a negative *R*_CT_ is a constant phase element (CPE) which accounts for the negative (*R*_CT_/CPE).^19–23^ The Warburg Impedance (*Z*_W_ = ¼*σ*_W_*ω*^−1^) (Ω), where *σ*_W_ = Warburg coefficient is identified by the straight line at 45°. It evaluates the species mass diffusion transfer from the bulk to the metal surface. This physical phenomenon depends on the speed with which a metal ion can be transferred from the surface to the bulk solution and how fast an electron acceptor can be transferred from the bulk structure to the metal surface to consume electrons.^24–27^ Since the Warburg portion of the curve presents a positive set of values (+*Z*′′) in a separate frequency range than the *R*_CT_/CPE element this indicated it was a separate component in the equivalent circuit diagram presented in Fig. 3.^28,29^

Charge Transfer Resistance (*R*_CT_) values for each powder were obtained from Nyquist plots like the one found in Fig. 3. Each sample was measured at its point of zero charge [Table 1]. Error values were determined for the pH_PZC_, and charge transfer values using each manufacturers equipment error specifications. For the Hanna Instrument (HI) pH meter/oxygen reduction potential (ORP) 2214 meter (ref. 30) is pH ± 0.4 °C^−1^. Temperature variation at each system's equilibrium temperature was pH ± 0.1 °C, giving an error of ± 0.04 pH for each titration run. Based on the documentation for the Gamry Instruments 600 Potentiostat/Galvanostat/ZRA^25,31,32^ error for the measured resistance was less than 1%. The values for each measured charge transfer resistance are presented in Table 1 and were calculated assuming a 0.9% error. Error values for the average primary particle diameter were calculated from results obtained by Leffler *et al.*^16^

**Table 1:** The average primary particle diameter, point of zero charge, charge transfer resistance and vendor information for the commercial powders used in the EIS work^16^

Average particle diameter variation/error (nm)	Point of zero charge (pH)	Charge transfer resistance^c^ (Ω)	Vendor information
5.3069 ± 0.0237	1.75 ± 0.04	70 ± 0.63	US3838^a^
12.689 ± 0.1819	2.48 ± 0.04	199 ± 1.791	US3490^a^
16.131 ± 0.534	3.04 ± 0.04	284.5 ± 2.561	US3492^a^
21.108 ± 1.225	4.25 ± 0.04	554 ± 4.989	US3493^a^
23.5379 ± 0.656	5.47 ± 0.04	273.7 ± 2.463	7910DL^b^
126.002 ± 0.760	7.15 ± 0.04	277.3 ± 2.496	US3411^a^
142.614 ± 1.064	7.19 ± 0.04	251.4 ± 2.263	US1152^a^

a U.S. Nanomaterial Research.

b Spring Sky Nanomaterials.

c This work.

**Fig. 4:**
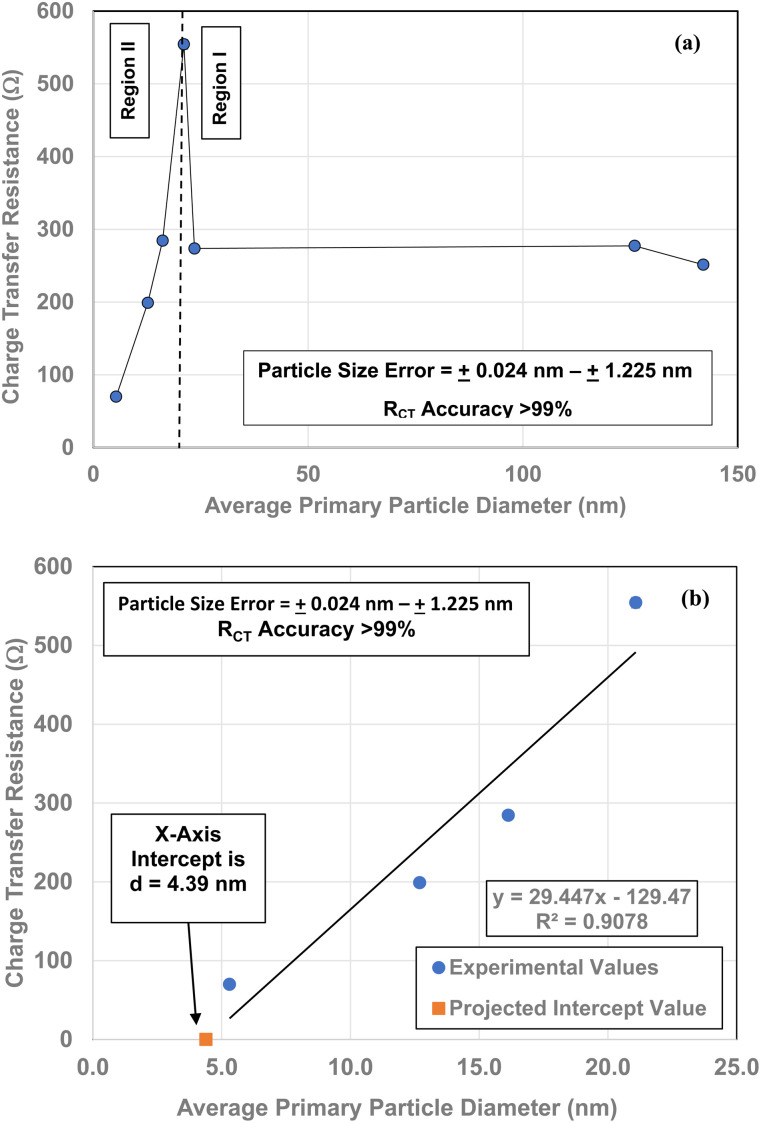
(a) A plot of all the powder population's charge transfer resistance (*R*_CT_/CPE) values against their average primary particle diameter for Regions I and II. (b) The *R*_CT_/CPE values for the linear portion of the curve (below *d* = 21.08 nm) in Region II. The plot was used to obtain a projected average particle diameter where *R*_CT_/CPE might go to zero.

Each of the measured powders *R*_CT_/CPE were then plotted against their average primary particle diameter in Table 1 [Fig. 4(a)]. Above a diameter of 23.54 nm, the charge transfer resistance remains essentially constant. There is then a significant increase in the resistance at *d* = 21.108 nm. Below *d* = 21.108 the *R*_CT_/CPE decreases almost linearly. Like the work by Salari *et al.*^3^ the projected average particle diameter in Fig. 4(b), where *R*_CT_/CPE goes to zero, has diameter of ∼4.39 nm. The results in Fig. 4(a) also suggest a surface structural change is responsible for the decrease in the materials *R*_CT_/CPE, not their average primary particle diameter since *R*_CT_/CPE remains constant as particle diameters increase from 23.54 nm to 142.614 nm. If this is the case, then it might be possible to obtain particles that present no *R*_CT_/CPE.

### 3.2 Test for dry magnetic separation

A thin layer of powder (*d* = 12.69 nm) was placed on the Grade N48 magnet and examined for any movement or change in shape due to the magnetic field. There was no apparent effect of the magnetic field on the powder. Therefore, it was determined that a dry separation method was not feasible, possibly because of the high surface charge and particle agglomeration of both the strong and weak diamagnetic particles which might have been preventing movement.

### 3.3 Initial results indicating an effect of a magnetic field on anatase titania in deionized water

It was found that when the anatase titania powder (*d* = 12.689 nm) in DI water was placed on the Grade N48 magnet, small thread/needle like shapes composed of particles formed. They were perpendicular to the base of the potash tube and remained during the time the tube sat on the magnet. When the tube was removed from the magnet, the threads/needles slowly fell over into the powder at the base of the tube and broke apart. This procedure was repeated multiple times, with the same result.

### 3.4 Magnetic settling in 0.01 N HCl

Magnetically separated particles in 0.01 N HCl exposed to ambient light [Appendix A.1] were found to partially settle. At the base of the Potash A tube on day 30 a powder ring was present indicating that a portion of the powder population had been forced out of the strongest region of the vertical magnetic field during settling [Fig. 15]. The powder remaining in solution at the end of the run, on day 43, was evenly dispersed in the 0.01 N HCl [Fig. 16]. The particles showed no evidence of the magnetic field affecting as to how they were distributed in solution. This suggested that a second variable was responsible for the dispersed particles remaining in solution [Fig. 16].

At the base of the Potash tube, before the solution was removed [Fig. 17], concentric powder circles were present. Between these circles and at the center of the smallest circle no powder was present. This indicated the patten in which the particles had been rejected from the magnetic field during settling. The clear regions demonstrated that particles dispersed in 0.01 N HCl were sufficiently diamagnetic to be affected by the Grade N48 magnetic field. After the removal and evaporation of the solution [Fig. 18] only one concentric powder circle remained.

### 3.5 Settling of anatase titania in deionized water with No magnetic field present

After 21 days of settling without the presence of a magnetic field, most of the fully dispersed particles [Appendix A.2, Fig. 19 and 20] were observed to have settled to the base of the Potash A tube. A small fraction of these particles though was evenly distributed in the DI water, when examined on days 26, 36 and 46. The density of these suspended particles in the solution remained constant until the end of the run at day 46. Based on the length of time the material had to settle, it became evident that these dispersed particles were permanently suspended in the DI water. As anatase titania is photoelectric, it was hypothesized that the surface charge, produced by exposure to ambient light, might be responsible for the continued suspension in the solution.

To test this hypothesis, a metal can was placed over the Potash A tube [Fig. 21] and then checked 10 days later. At this time the water was significantly clearer, due to the settling of a large percentage of the suspended particles. At the end of the run, with the metal can in place (day 41) the DI water was found to be completely clear [Fig. 22]. The only remaining particles not settled were found as a residue on the side of the Potash A tube. The entire experiment, without and with a metal cup excluding ambient light, extended over 87 days [Fig. 23].

### 3.6 Magnetic separation in deionized water with all ambient light excluded

Magnetic separation in deionized water with ambient light excluded made it possible to obtain only particles susceptible to the magnetic field. Appendix A.3 demonstrates the separation process over the 15 days period which was determined to be required for a full separation. An interesting note, Fig. 24 demonstrates a region of clear DI water at the top of the tube on day 4, which had filled in with particles by day 8 Fig. 25. This is further evidence that the more strongly diamagnetic particles were pushed upward into this region, and sitting higher in the magnetic field, than those below them. This mimics what was seen for superconducting powders under dry conditions in a cooling chamber.^17^Fig. 26 demonstrates that particles susceptible to levitation are most concentrated in the strongest region of the magnetic field.

A series of runs on the same powder population were executed. This was accomplished by removing the magnetically levitated particles at the end of each run using the 200 μL Gilson pipettor. The settled particles were redispersed by recapping and agitating the tube. The tube was then set back on the magnet and covered by the cylindrical cardboard box to exclude all ambient light. The last run was determined [Fig. 27] when the fully dispersed particles had settled out of the DI water leaving it clear. This typically took approximately an hour. Any remaining particles were located at either the bottom of the tube or as residue on its walls. The particles remaining as residue also indicated that their surfaces likely possessed a high static charge. This is interesting, as there was no available light to engage electron production at the surface through the photoelectric effect.

**Fig. 5:**
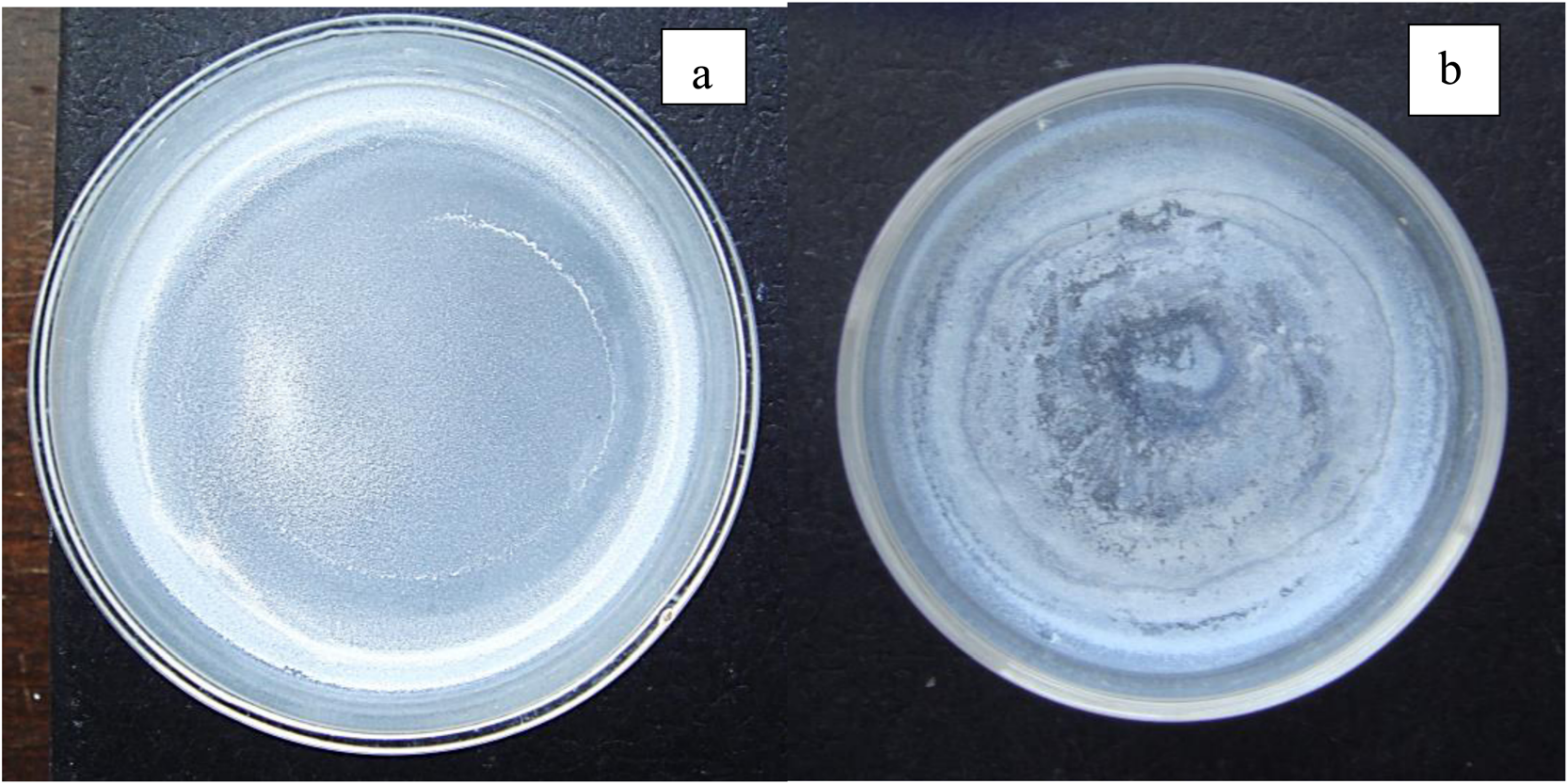
(a) After drying the parent population remained a bright white. (b) The magnetically separated powder after drying was sky blue.

An examination of the parent population, from which the magnetically separated powder was obtained, revealed a significant difference in their color [Fig. 5(a) and (b)] when dried. The parent population remained bright white, indicating it had a larger bandgap than the magnetically separated powder which exhibited a vibrant sky-blue color.^33^ Since band gap is directly related to the material's bond length^34^ this indicates that the magnetically separated particles have longer global bond lengths than the parent particle population. Therefore, this difference in colors between the two samples confirmed that a magnetic separation had occurred.^16,35^

### 3.7 Effect of no ambient light or magnetic field present on the magnetically separated anatase titania particles

The magnetically separated particles, in the same DI water segregation occurred, were placed under the metal can excluding all ambient light with no magnetic field present. This resulted in the particles agglomerating and settling to the base of the potash tube. These agglomerated magnetically separated particles, in DI water, were then placed back onto the Grade N48 magnet used to levitate them. It was found that they were now unaffected by the magnetic field. It was also noted that that there was either little or no DI water in the interstitial regions between these interior particles of the agglomerated particles. This points to the importance of the presence of water fully surrounding each particle to effect magnetic levitation.

**Fig. 6:**
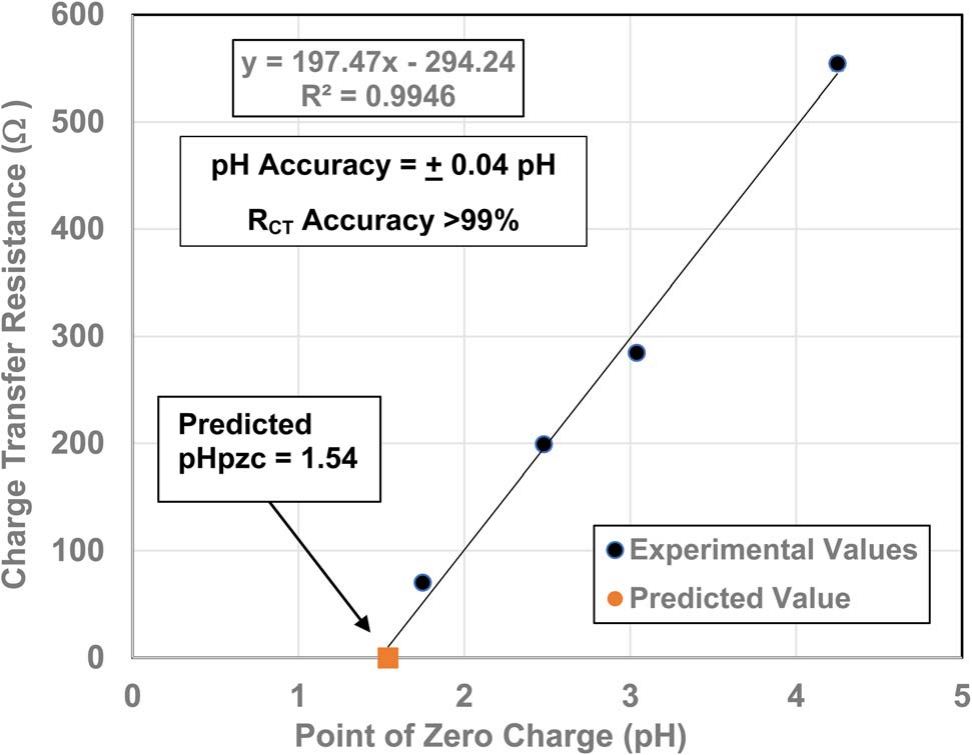
Projected point of zero charge for the material at which zero electrical resistance might occur.

When the agglomerated particles were re-exposed to ambient light with no magnetic field present, the particles slowly broke away from the agglomerate and returned to solution. The process of deagglomeration took approximately two months. This suggests that the photoelectric property of the material may play a critical role in allowing anatase titania to remain suspended in solution indefinitely outside a magnetic field [Fig. 6].

### 3.8 Charge transfer resistance values for magnetically separated anatase titania particles

**Fig. 7:**
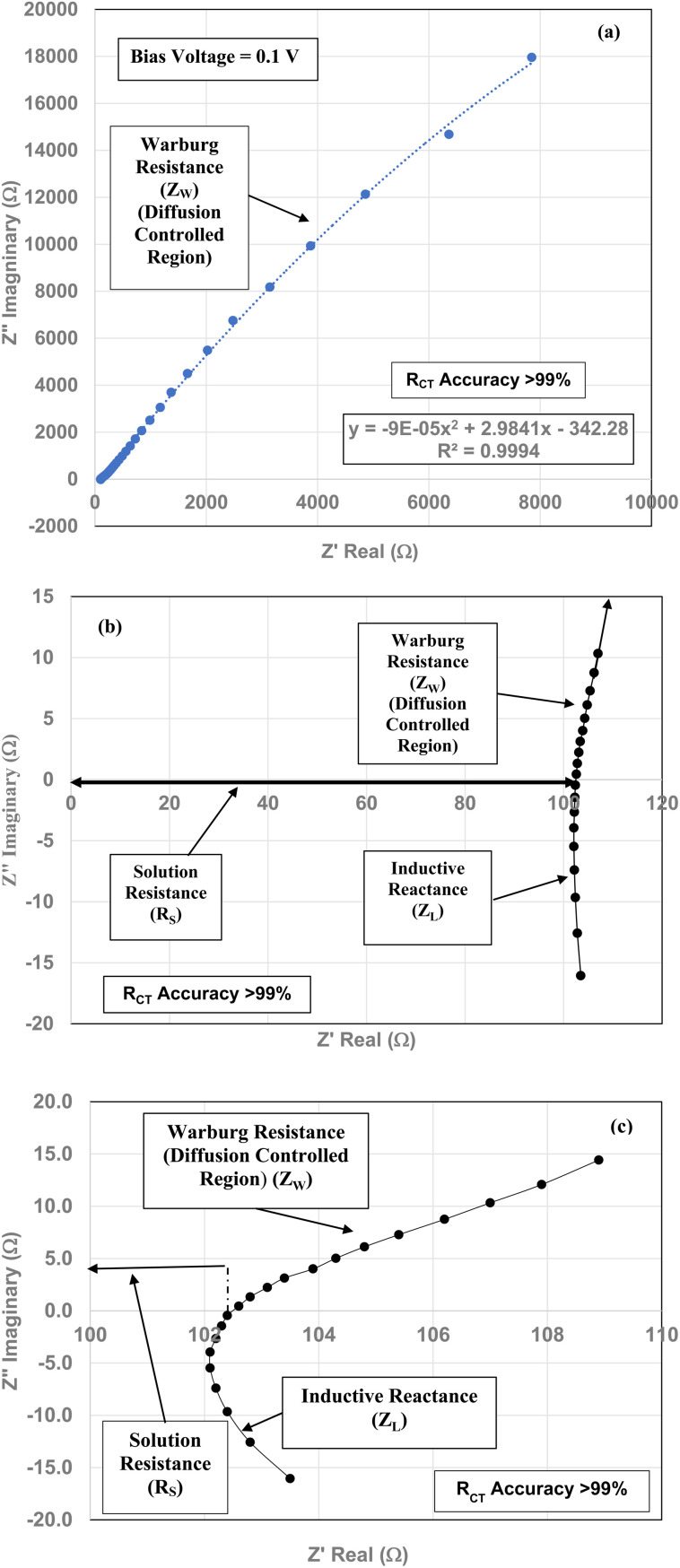
(a) The Nyquist plot for the magnetically separated pellet at a 0.1 V bias across the full frequency range. (b) The expanded portion of the Nyquist plot above and below the real axis (*Z*′). The portion of the plot which extends from the origin to the first point where the highest frequency point intersects the real axis (*Z*′) is the solution resistance (*R*_S_). The sum of the inductive reactance (*Z*_L_) and Warburg resistance (*Z*_W_) curves are present above and below the *Z*′ axis. (c) The expanded portion of the Nyquist plot above and below the *Z*′ axis at the highest frequencies. Only the solution resistance (*R*_S_) inductive reactance (*Z*_L_) and Warburg resistance (*Z*_W_) are present. There is no semi-circle for the *R*_CT_/CPE present in any portion of the Nyquist plots.

To obtain the projected pH_PZC_ value for the magnetically separated particles, a plot of the particle sizes from 5.31 nm to 23.54 nm against their pH_PZC_ values [Table 1] was made. A regression curve was then fitted to the plotted values. Using the equation of the fitted regression curve, a pH_PZC_ = 1.54 was calculated at a value of *R*_CT_ = 0.0 Ω [Fig. 7]. The correlation value for the fitted curve was *R*^2^ = 0.9946, indicating that the projected pH_PZC_ value at *R*_CT_ = 0.0 Ω was highly reliable.

**Fig. 8:**
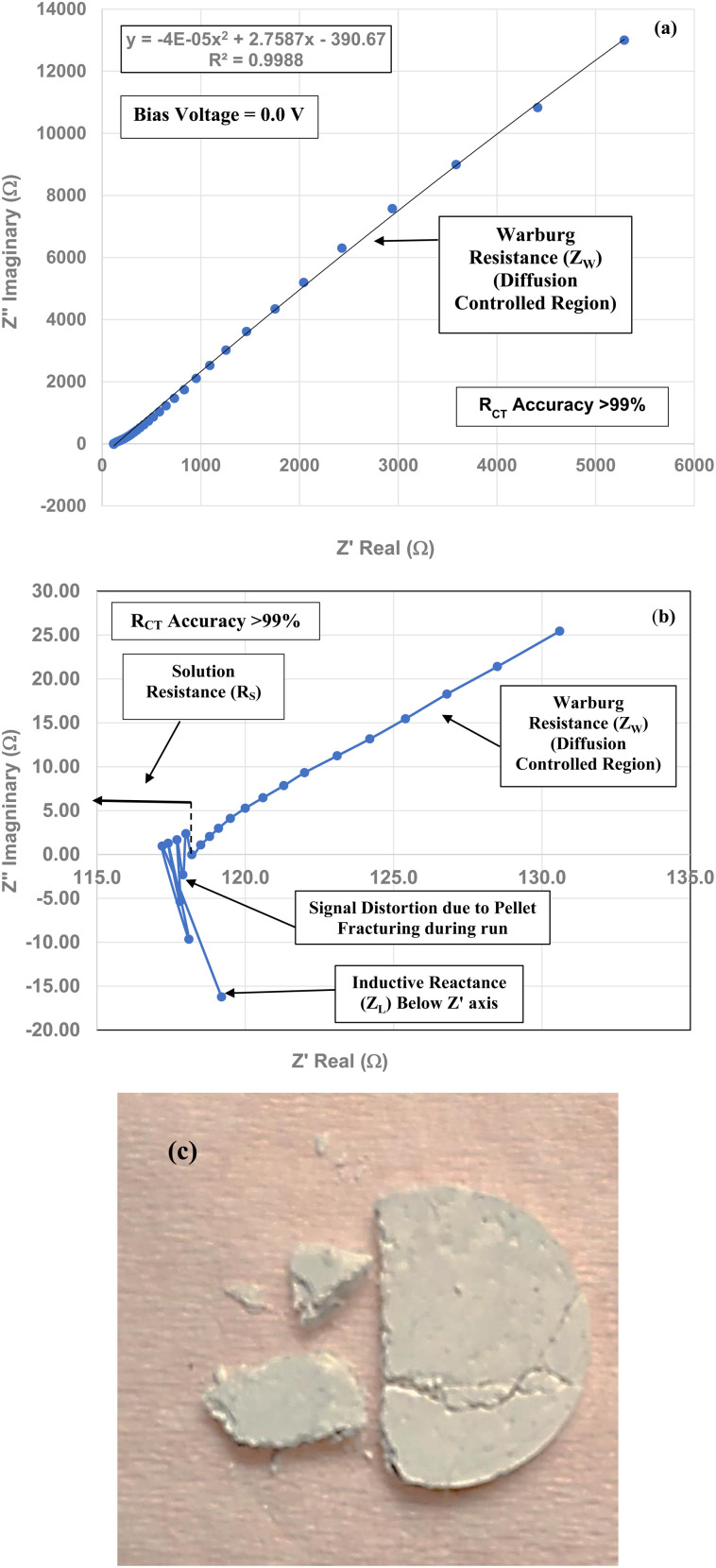
(a) The Nyquist plot for the full frequency range of the run for the magnetically separated powder pellet at 0.0 V bias. (b) The high frequency range for the Nyquist plot shows only *R*_S_, *Z*_L_, and *Z*_W._ The signal distortion was caused by the pellet fracturing during this second run at 0.0 V bias. (c) The magnetically separated pellet that fractured during the run at 0.0 V bias, causing the signal distortion.

**Fig. 9:**
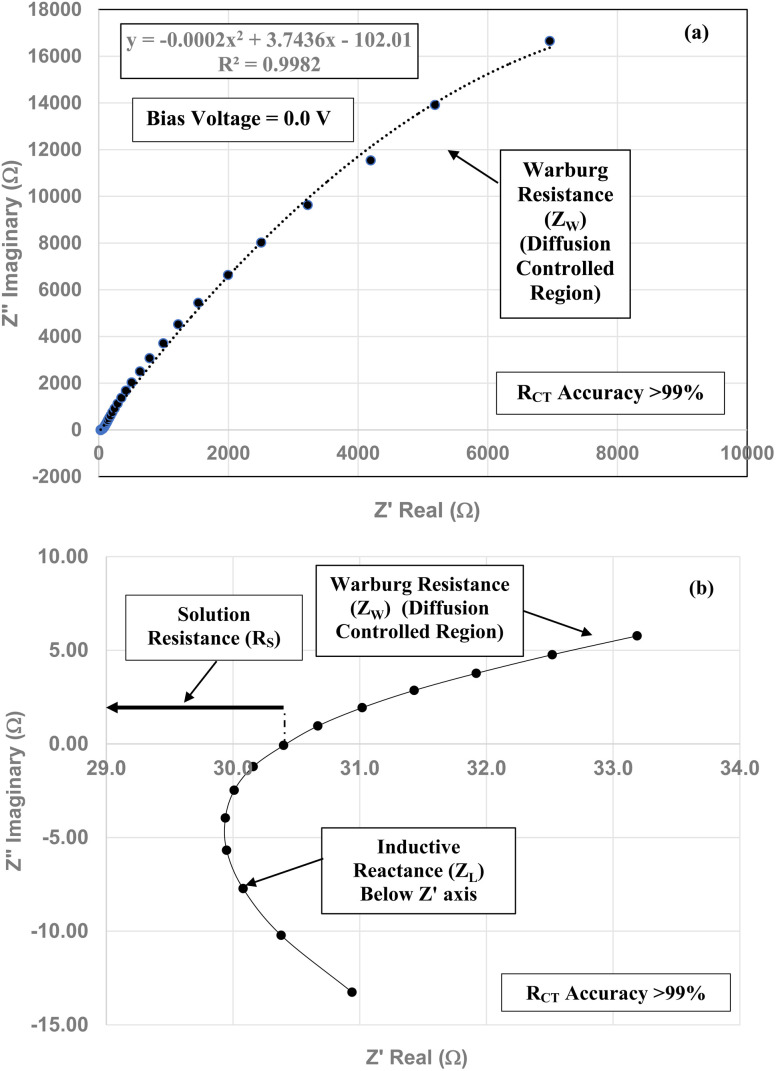
(a) The Nyquist plot of the unloaded Teflon coated Pt clip/electrode possesses the same characteristic curve as Fig. 7(a)–(c) and 8(a) and (b) over the identical frequency range. (b) Displays solution resistance (*R*_S_) and the sum of the values for the Warburg resistance above the *Z*′ axis and the induction portion of the curve below the *Z*′ axis.

Three EIS runs were performed to obtain charge transfer results [Nyquist plots]. The first run [Fig. 7(a)–(c)] was for the magnetically separated particle pellet under 0.1 V bias. A second run was performed at 0.0 V bias [Fig. 8(a) and (b)]. In Fig. 8(b) there is signal distortion due to fracturing of the pellet [Fig. 8(c)] during the run. After completing these two runs, a third run was performed at a 0.0 V bias, where no sample was present in the Pt clip [Fig. 9(a) and (b)]. This run was executed so that the curves with and without a sample present could be compared.

**Fig. 10:**
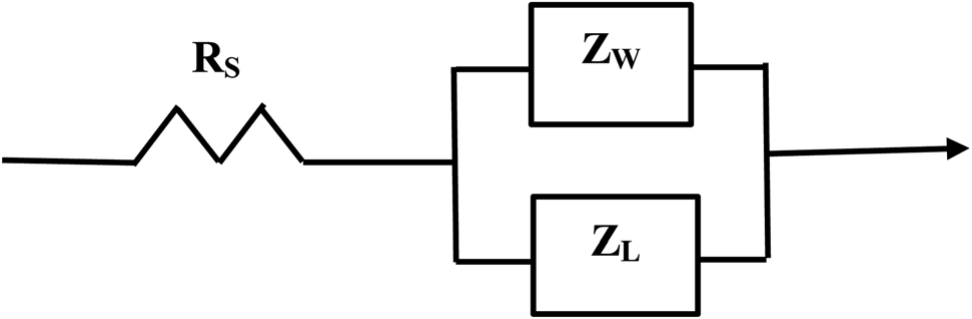
The equivalent circuit of the curves in Fig. 7(a)–(c) through Fig. 9(a) and (b). Solution resistance (*R*_S_) is measured from the origin to where the highest frequency point is recorded intercepting the real axis (*Z*′). Beyond *R*_S_ on the *Z*′ axis are the summed values of *Z*_L_ and *Z*_W_ across the same frequency range.

Above the *Z*′ axis the curve is at 45°, indicative of Warburg impedance.^36–38^ There are no semi-circles indicating *R*_CT_/CPE are not present as it was in Fig. 2. Therefore, this pellet exhibits no *R*_CT_/CPE (*i.e.*, bulk electrical resistance/impedance). The equivalent circuit for these three runs, Fig. 7(a)–(c), 8(a), (b) and 9(a) and (b), is presented in Fig. 10.

A Nyquist plot where a sample was not present (*i.e.* an unloaded system) was made by Lin *et al.*^39^ The plot they obtained is identical in configuration to those in Fig. 7(a)–(c) through Fig. 9(a) and (b). This further supports the analysis of these three sets of figures that the sample under test demonstrated no *R*_CT_/CPE (*i.e.* bulk electrical resistance) [Fig. 10].

### 3.9 Determination of average primary particle diameter for magnetically separated anatase titania

**Fig. 11:**
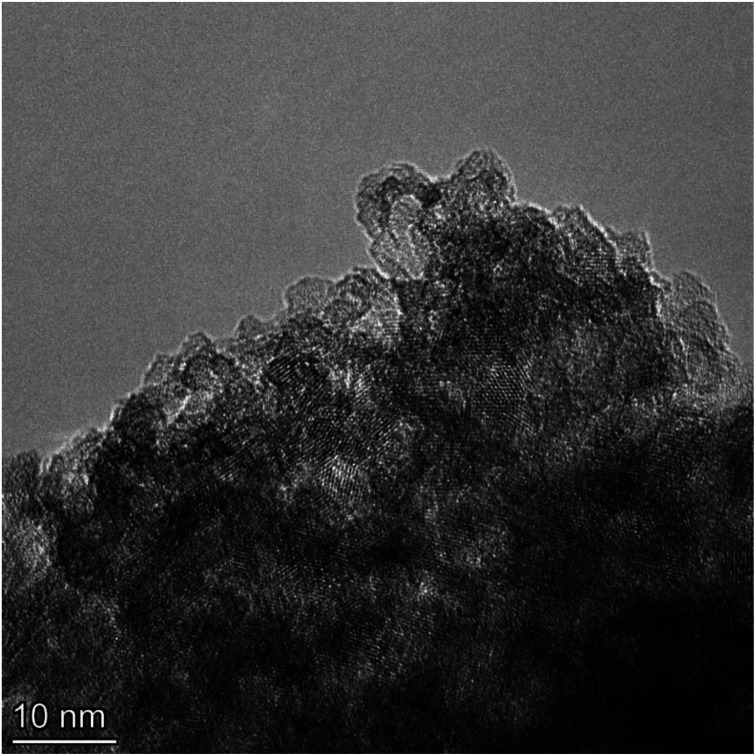
A TEM micrograph of magnetically separated particles from the parent population with an average primary diameter of 3.30 nm.

Multiple TEM micrographs were taken of the magnetically separated particles [Fig. 11]. Using the method developed by Leffler *et al.*,^16^ both the spherical and equivalent spherical diameters were measured by hand from the TEM micrographs. It was possible to clearly identify 68 values, where magnetically separated particle diameters ranged from 1.739 nm to 4.56 nm. This gave an average primary particle diameter of 3.30 ± 0.50 nm. This value is 1.09 nm smaller than the average diameter predicted in Fig. 4(b) (*d* = 4.39 nm), but still within the full range of particle sizes identified.

In addition, the difference between the average primary particle diameter for the parent population was *d* = 5.31 nm. The magnetically separated average primary particle was *d* = 3.30 nm, ∼2 nm smaller than the parent population. This, in conjunction with the different color of the powder (*i.e.* sky-blue) also confirms that a separation occurred between the magnetically susceptible and non-magnetically susceptible particles.

### 3.10 Correlation of *R*_CT_/CPE and point of zero charge

**Fig. 12:**
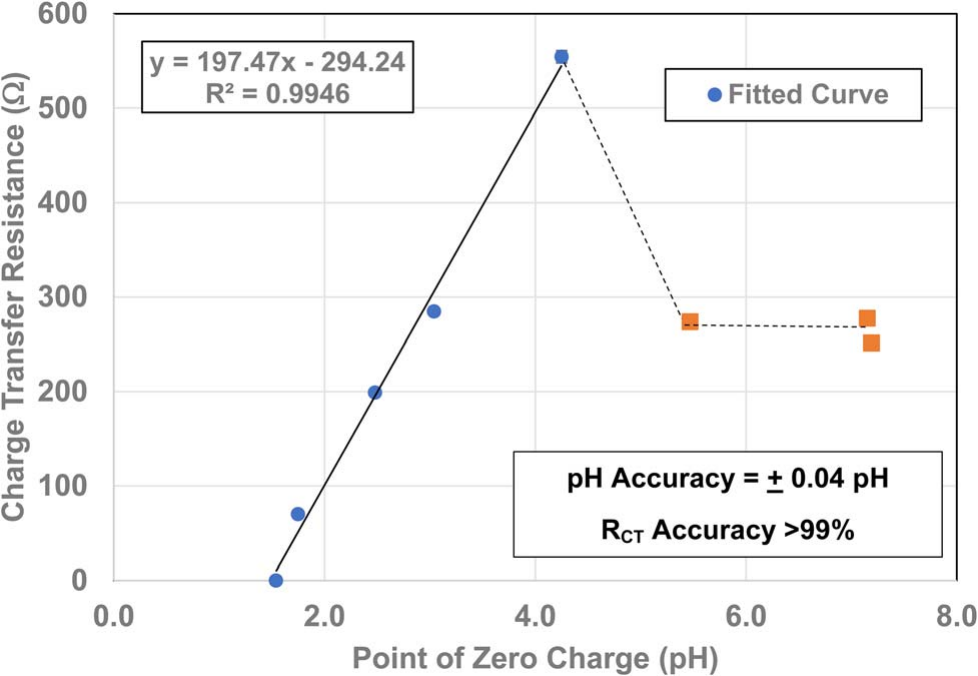
A plot of each powder population's point of zero charge^16^ against its *R*_CT_/CPE. The fitted curve extends from pH_PZC_ values 1.54 to 4.25.

To determine if a relationship exists between the materials point of zero charge and their *R*_CT_/CPE, these two values were plotted against one another. This was possible as all the materials measured came from the same powder populations.^16^ Therefore, their average primary particle diameters are identical allowing for a direct correlation between the two properties. The plot [Fig. 12] demonstrates that there is a simple linear relationship between these two values from pH_PZC_ = 4.25 down to pH_PZC_ = 1.54. Above a pH_PZC_ = 4.25 the charge transfer resistance decreases to an average value of 267.5 Ω. Beyond a pH_PZC_ = 5.47, *R*_CT_/CPE values remain constant. These results indicate a direct correlation between these two properties and suggests that the underlying changes in surface structure/properties are responsible for changes in PZC and *R*_CT_ values

### 3.11 Correlation of *R*_CT_/CPE and band gap

**Fig. 13:**
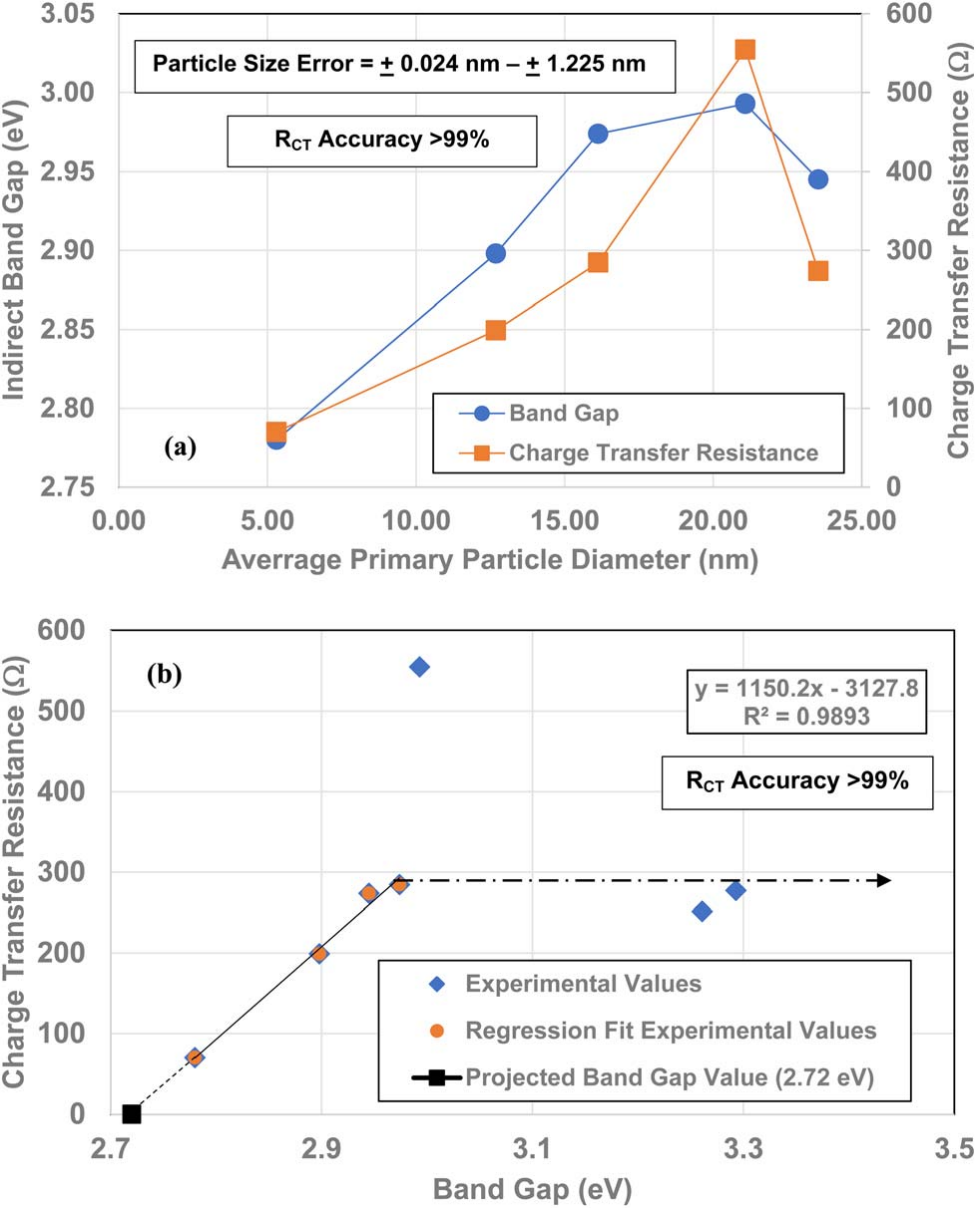
(a) Bandgap^16^ and *R*_CT_/CPE values plotted against their average primary particle diameter. (b) Bandgap values^16^ plotted against their *R*_CT_/CPE. The projected band gap value of 2.72 eV is for *R*_CT_/CPE = 0.0 Ω.

Leffler *et al.*^16^ demonstrated that the band gap for all seven powder populations correlates directly with the change in the materials point of zero charge. Therefore, to determine if this relationship holds for the *R*_CT_/CPE and band gap values from 5.31 nm to 23.54 nm against their average primary particle diameters were plotted alongside each other. Based on both curves there is a strong visual correlation between the two variables [Fig. 13(a)].

When the band gap values are plotted against their *R*_CT_/CPE [Fig. 13(b)], there is a highly correlated (*R*^2^ = 0.9893) linear relationship between them from particle sizes *d* = 5.31 nm to 16.31 nm and *d* = 23.54. The particle diameter where there is an anomalous increase *R*_CT/_CPE is at *d* = 21.08 nm (*ρ* = 554.3). This is most likely the average primary particle size where the surface structure is transforming to the bond lengths and atomic positions where the *R*_CT_/CPE (*d* = 23.54–142.02 nm) remains constant.

### 3.12 Correlation of *R*_CT_/CPE with *a*/*b* and *c* lattice parameters

**Fig. 14:**
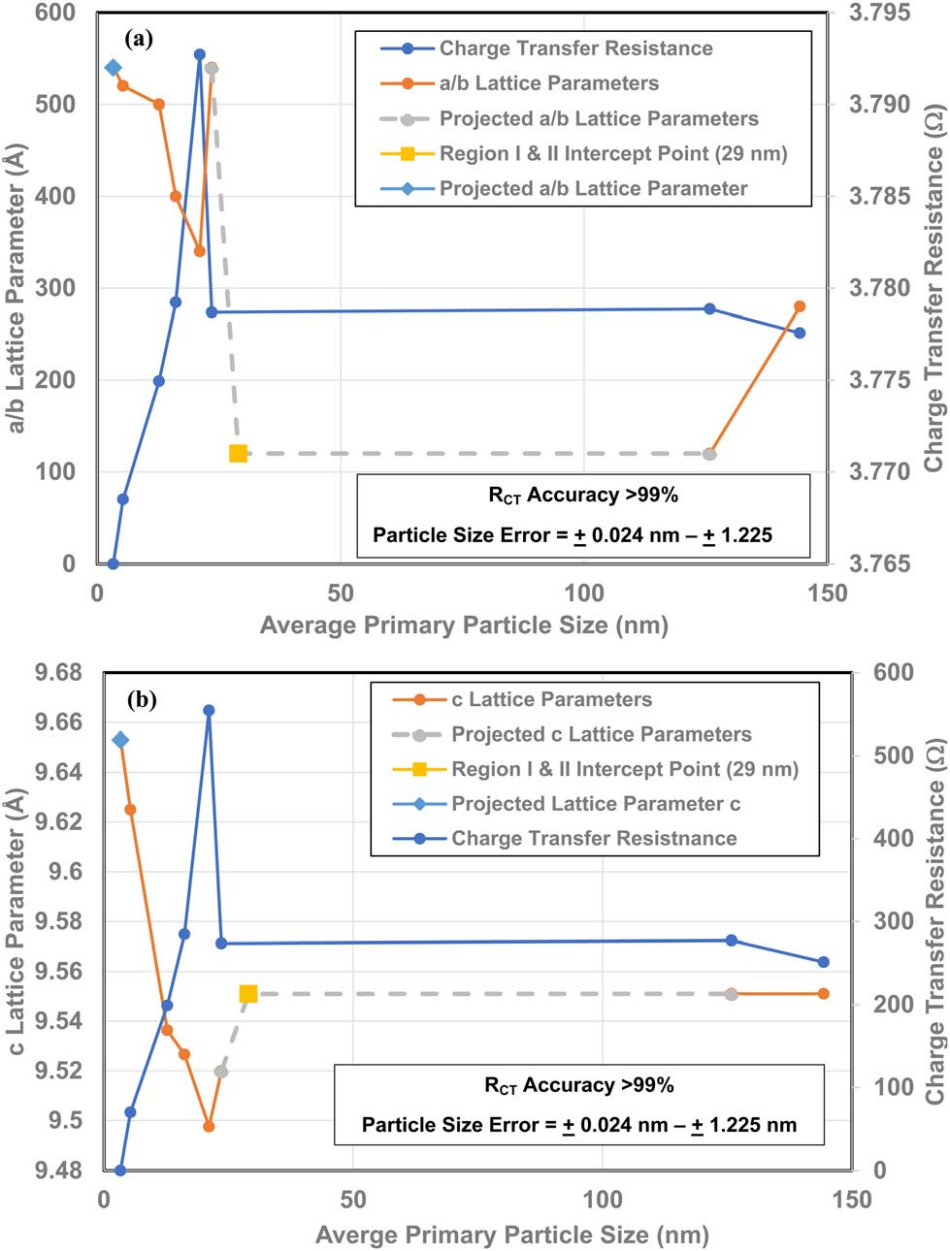
(a) Direct comparison of the *R*_CT_/CPE and *a*/*b* lattice parameters^16^ for all the sample populations measured. (b) A direct comparison of the *R*_CT_/CPE and the c lattice parameters^16^ for all the samples measured.

There is a direct linear correlation between each material's *R*_CT_/CPE and its band gap [Fig. 14 (a) and (b)]. As band gap is a measure of the changes in the global surface bond lengths a set of plots for the *a*/*b* and *c* lattice parameters^16^ alongside the *R*_CT_/CPE were made. As all but one data point is for the same average primary particle diameters, the plot is a direct correlation between these two values [Fig. 14(a) and (b)].

The value at *d* = 29 nm is the intercept point for Regions I and II.^16^ For values with particle sizes greater than *d* = 29 nm, are in Region I where neither the measured properties nor the surface structure changes. This makes it possible to extrapolate from this data point to a measured value (*d* = 125.85 nm) in Region I. In Region II, each of the *R*_CT_ curves very closely parallel the plots for *a*/*b* and *c* lattice parameters, allowing the extrapolation from the value at *d* = 23.54 nm to the intercept point at *d* = 29 nm.

It is evident from Fig. 14(a) and (b) that below a particle diameter of *d* = 21.08 nm both the *a*/*b* and *c* lattice parameters are expanding. This indicates that that as the *R*_CT_/CPE decreases below a diameter of 21.08 nm, the Ti and oxygen atoms are moving away from each other. Therefore, in conjunction with findings by Leffler *et al.*^16^ it indicates that the surface is shifting towards the internal bulk structure bond lengths and atomic positions of each crystallite.^40^

## 4 Discussion

From the results in Section 3.2 it is evident dry magnetic separation, which has been used on high temperature superconducting powders,^17^ does not work on the anatase titania particles. This process uses dry high temperature superconducting powders, levitating them above a magnet in a cold chamber below the materials transition temperature (*T*_C_). Whereas the method developed for anatase titania magnetic separation requires an aqueous environment [Sections 3.3 through 3.6], at ambient temperature and pressure. This pointed to a different mechanism responsible for the magnetic separation. Once the bulk of the water from around the particles was removed due to agglomeration [Section 3.7] they would not levitate. Therefore, it became evident that each of the magnetically separated particles required water to completely cover its surface.

Anatase titania is weakly diamagnetic, therefore all the particles surface orbitals (*i.e.*, currents) are paired, but were unable to affect levitation of the agglomerated material. In addition, at ambient temperature and pressure only single electrons were present in the conduction band of this material. This is evident from the EIS method used to measure *R*_CT_/CPE for each of the samples in Fig. 2, 4(a) and (b). All the samples were measured on the same EIS system [Section 2.2] at ambient temperature and pressure, under identical conditions, save PZC.

Both low and high temperature superconductors^41–43^ and the magnetically separated anatase titania particles [Fig. 7(a)–(b), 8(a) and (b)] conduct electricity without resistance. Yet only the first set, the low and high temperature superconductors possess Cooper pairs. Cooper pairs form just below their *T*_C_ at which the material enters the superconducting state.^44,45^ They have either a 1 or 0 spin,^46^ indicating they are perfectly diamagnetic.^47^ Yet the surface currents (*i.e.*, orbitals) at the anatase titania's agglomerated particle surfaces were unable to levitate the material. Therefore, this also suggests that in the low and high temperature super conductors surface currents may not be sufficient to produce the Meissner Effect (*i.e.*, magnetic levitation).

Unpaired electrons are present, in low and high superconductors, in their conduction band, above the materials *T*_C_.^48^ When placed in a magnetic field, above the materials *T*_C_, the field lines pass completely through these superconductors.^49^ Below the transition temperature though, Cooper pairs form,^44^ and the magnetic field is completely excluded from the superconducting material.^49,50^ This suggests that the formation of Cooper Pairs may be the critical factor in fully expelling the magnetic field lines in low and high super conductors. They may act in the same manner as fully filled orbitals (*i.e.*, diamagnetic) at the surface but also located throughout the superconductor's entire structure within its conduction band network.^51^ Their presence and position throughout the structure might be why the magnetic field is expelled from both the surface and interior of the superconducting material.

The first part of the mechanism leading to the magnetic separation of the anatase titania particles may be due to electric dipole–dipole interaction between the DI water and the anatase titania particles. Water has a very strong electric dipole moment (p) due to its bent structure.^52^ While micron sized particles of TiO_2_ have a weak electric dipole moment of 6.33 Deby.^53^ For nanoparticles of TiO_2_ with an average diameter *d* ∼3.0 nm though, Yan *et al.*^54^ demonstrated a dramatic increase in their electric dipole. They determined the particles had a time-averaged electric dipole moment fluctuating between 40 to 80 Deby at 273 K, significantly greater than the macroscopic particles.^54^ To illustrate how strong this electric dipole for the nanoparticles of TiO_2_ is, CsI is considerably above the average electric dipole moment at 11.69 Deby.^55,56^

This significant increase in the electric dipole moment for TiO_2,_ with decreasing particle size, would then dramatically increase the interaction between the particles and water molecules. Also, as this is an aqueous environment water might also easily orient itself to arrange a three-dimensional network of molecules and particles of alternating charge, akin to a very dilute solution gelation structure.^57^ This might also explain why a small fraction of particles remained in solution, even without the presence of a magnetic field when exposed to ambient light [Section 3.4]. Since the agglomerated particles returned to solution as individual particles when re-exposed to ambient light, this suggests the material's photoelectric property may also play a part in the electric dipole–dipole interaction with water molecules.

The proximity of water molecules and anatase titania particles might then have facilitated the second part of the possible mechanism, the formation of a global magnetic dipole (*μ*_B_) moment through magnetic dipole–dipole coupling (*i.e.*, addition).^58^ This magnetic dipole–dipole interaction is present in magnets, where the sum of the magnetic dipoles in each domain are responsible for the strength of its global magnetic field.^59^ In the magnetic separation method developed, it appears to require the presence of magnetic dipole moments from multiple sources, not only the surface orbitals (*i.e.*, surface currents) of the superconducting material. This is again underscored by the fact that the agglomerated particles would not levitate in the magnetic field by themselves, as water surrounding all the particles was missing. It might also explain why the dry powder separation method did not work. There was no water magnetic dipole to interact with both the oxygen and titanium atoms.

While a diamagnetic material, such as water, has no magnetic dipole moment outside of a magnetic field,^60^ it does develop one when placed in a magnetic field.^61^ Anatase titania is also a diamagnetic material, indicating it would have no magnetic dipole moment. But Mombru *et al.*^62^ demonstrated that the individual atoms within the structure do possess significant magnetic dipole moments. They determined that the atoms with the strongest magnetic dipole moments (*μ*_B_) are the Ti atoms, for all structures of titania, when they are of the size of quantum dots with a diameter at ∼5 nm. Their *μ*_B_ = 4.0 individually. Values for the oxygen atoms possess a lower range of values, from *μ*_B_ = 0.02 to *μ*_B_ = 0.98.

Surface structural changes may also have contributed to a change in the particle's global magnetic dipole moment. Leffler *et al.*^16^ demonstrated that as the primary particle size of anatase titania particles above an average primary particle diameter of ∼29 nm, oxygen atoms sit above the Ti atoms. In addition, the electrons transferred to the oxygen atoms bow back, shielding the Ti atoms and possibly their surface orbitals, situated below them. This may have resulted in the cancellation of the Ti atoms magnetic dipole moments^63^ nearest the surface. If so, this would have reduced the overall strength of the surface magnetic dipole–dipole interactions (*i.e.*, addition).

With decreasing primary particle size, the Ti atoms would have moved upward to the same level as the oxygen^16^ on the magnetically separated particles surface. In addition, the electrons shielding the Ti might have completely retracted back to surround only the oxygen. This may have resulted in their Ti atoms magnetic dipole moments no longer being cancelled by the shielding oxygen orbitals which had been situated above them. The unshielded Ti magnetic dipole moment^62^ at the surface might then have coupled with both those of the oxygen and water magnetic dipole moments when placed in a magnetic field. This coupling of all the individual magnetic dipole moments might then have formed a global magnetic moment strong enough to levitate the individual particles allowing for the magnetic separation to occur.

Fig. 7(a)–(c) through Fig. 9(a) and (b) demonstrate that there are no semicircles present, indicating *R*_CT_/CPE was not detected in either the magnetically separated anatase titania particles or the Pt electrode. Only solution resistance (*R*_S_), inductive reactance (*Z*_L_) and Warburg reactance (*Z*_W_) (diffusion-controlled resistance) are present in each of these Nyquist plots. The realistic minimum impedance limit measurable for a Gamry system is 10^−6^ Ω.^64^ Platinum has an electrical resistance of 10.6 × 10^−8^ Ω m (ref. 65) which is below the minimum detection limit of the Gamry EIS system used making it unlikely it was a component of the *R*_S_ measured. Therefore, if the magnetically separated particles have any electrical resistance (*i.e. R*_CT_/CPE), they too are most likely below the detection limit of the EIS system [Section 2.2] employed.

EIS has been used successfully to characterize the high temperature superconducting system YBa_2_Cu_3_O_7−*δ*_/RbAg_4_I_5_. Two Nyquist plots for this material were recorded below its transition temperature (*T*_C_ = 92 K), and one above it. Each of the Nyquist plots exhibited semi-circles, indicating that a charge transfer resistance (*i.e.*, bulk electrical resistance) was present. Below the materials *T*_C_ the average *R*_CT_ are between 180 × 10^−6^ Ω cm^2^ to 190 × 10^−6^ Ω cm^2^. Above the *T*_C_ the *R*_CT_ increased to approximately 140 Ω cm^2^.^43^ Their work suggests that had *R*_CT_/CPE been present within the equipment's range of sensitivity^66^ the EIS used would have measured it in the magnetically separated particle pellet and Pt electrode.

From Fig. 12, it is evident that there is a direct linear relationship between the material's point of zero charge and *R*_CT_/CPE from pH_PZC_ = 4.25 down to pH_PZC_ = 1.54. This demonstrates that the surface structural/property transformation mechanism responsible for the shift in PZC values^16^ is also the underlying mechanism responsible for the decrease in the materials *R*_CT_/CPE at/and below an average primary diameter of 21. 08 nm [Fig. 4(a)].

Fig. 13(a) demonstrates a direct visual correlation between the indirect bandgap values and *R*_CT_/CPE from the average primary particle diameter of 5.31 nm to 23.54 nm. A plot of each material's band gap values against their *R*_CT_/CPE is presented in Fig. 13(b). The plot reveals a charge transfer resistance anomaly in the sample with a diameter 21.08 nm. The fitted line consists of particle diameters from 5.31 nm to 16.13 nm and 23.54 nm. At a diameter of 21.08 nm the charge transfer resistance increases dramatically by almost 270 Ω (*ρ* = 554.3 Ω), even though the bandgap value increased by only 0.019 eV. Then little more than an increase of 2.49 nm in the diameter of the next larger particle size (*d* = 23.54 nm), the materials *R*_CT_/CPE drops to 274 Ω and remains essentially constant up to the largest particle diameter of 142.02 nm. This suggests that at *d* = 21.08 nm the surface is in the process of shifting to the surface structure found at, and above *d* = 23.54 nm.^16^

The expansion of the *a*/*b* and *c* lattice parameters [Fig. 14(a) and (b)] below an average diameter of 21.08 nm indicates surface atoms are moving away from each other. While at the same time the metal atoms are moving upward toward the surface.^16^ This suggests that the controlling mechanism for *R*_CT_/CPE is linked directly to changes in the materials surface structure, not the bulk. The particles with diameters at and above 23.54 nm also support this contention, as their charge transfer resistance [Fig. 4(a), 12, 13(b), 14(a) and (b)] remains essentially constant.

For electrons to move from one particle to another, as occurs in Fig. 7(a)–(c) and 8(a) and (b), the conduction band must possess an opening at the surface. Above a diameter of 29 nm^16^ the oxygen atoms are situated above the level of the Ti atoms at the surface and are drawn up and over them.^67^ The electrons (*i.e.*, orbitals) transferred from the Ti atoms to the oxygen bow back toward the Ti atoms partially shielding them.^16^ These shielding orbitals may also lie across the conduction band opening at the surface.

These shielding orbitals might also act as a potential energy barrier to electrons emerging at the surface from the conduction band. As all electrons possess a spin, they create a magnetic moment. The shielding orbitals above the Ti atom and the electrons leaving the conduction band at the surface, might then interact in the manner of two magnets of like poles. This would probably be present at all the particle surfaces for each pellet measured.

Leffler *et al.*'s^16^ work suggests that as the average primary particle diameter decreases toward *d* = 3.30 nm, the shielding electrons retract back until they are only present around the oxygen atoms. This might have resulted in the potential energy barrier completely disappearing from the path of the electrons emerging from the opening of the conduction band at the particles surface. If so, this mechanism would also have occurred on the surface of all the adjacent particles throughout the pellet. That would make it a simple matter for the electrons exiting the conduction band at the surface of each particle to move easily to the next opening of the closest conduction band opening on an adjacent particle surface.

The last factor which might have affected the materials *R*_CT_/CPE was a possible electro-static interaction between the potential energy barrier and a single electrons electrical charge. This might not be a factor if the potential energy barrier is not present due to the orbitals having retracted around the oxygen atom. But if some of the barrier is present, measurement at a material's point of zero charge would result in the particle's net surface charge being neutralized, possibly eliminating this interaction. This might also have reduced some of the resistance for each of the powder samples measured as well. It might also explain why the *R*_CT_/CPE measured for the magnetically separated sample was below the detection limit of the EIS system used.

There is a discrepancy between the low and high temperature superconductors, and the magnetically separated particles. Several high temperature superconductors exhibited very low, but measurable *R*_CT_/CPE (Nyquist plots),^41,43^ while the magnetically separated anatase titania particles did not. Appendix A.4 discusses this discrepancy and the possible insights it might afford into the BCS model.

## 5 Conclusions

A series of EIS measurements for anatase titania same weight pellets with decreasing average primary particle sizes were measured. Plots of their *R*_CT_/CPE values projected a particle size of 4.39 nm where this value might go to zero. To obtain this material a magnetic separation method was developed.

EIS measurements were then conducted on the magnetically separated material at the same weight pellet form, in an electrolyte at the material's projected point of zero charge. These tests were performed on the pellets at bias voltages of 0.1 V and 0.0 V. The Nyquist plots for both bias conditions determined that only three types of reactance (*i.e.*, active resistance) were present. These were, solution resistance inductive reactance, most likely caused by the Pt electrode and Warburg resistance (electrolyte diffusion-controlled region). For the unloaded run Warburg resistance most likely occurred at the Pt electrode's surface. No semi-circles were observed in any of the runs where the pellet was in the circuit, indicating that *R*_CT_/CPE (*i.e.*, bulk structure electrical resistance) was not present in the magnetically separated particles under test, indicating the material was superconducting.

## 6 Future work

The one drawback of the separation method developed for this work is the removal of the particles from the aqueous environment. This process requires multiple steps to obtain dry powders. A possible method for future work was developed by Yang *et al.*^68^ They used a low power laser to create convection currents in an aqueous solution. The nanotubes then impacted and adhered to a small bubble in the aqueous solution, in the same manner used in flotation methods to obtain, concentrate, and extract valuable ores and coal from the parent population.^18^

The advantages the method developed by Yang *et al.*^68^ is that it can be used as a bench top system to determine the ideal conditions under which the anatase titania and/or other magnetically separated materials can be harvested using full scale flotation methods. Since anatase titania possesses a positive surface charge,^16^ and air bubbles typically have a negatively charged surface,^69^ attachment and flotation are favored. An added advantage is that this method would also reduce and/or eliminate any possible contamination that the method used in this work may have caused.

## Data availability

All data is available on request

## Conflicts of interest

All sources of funding have been declared and there are no conflict of interest in this manuscript.

## A Appendices

### A.1 Magnetic separation of anatase titania particles exposed to ambient light in 0.01 N HCl

**Fig. 15:**
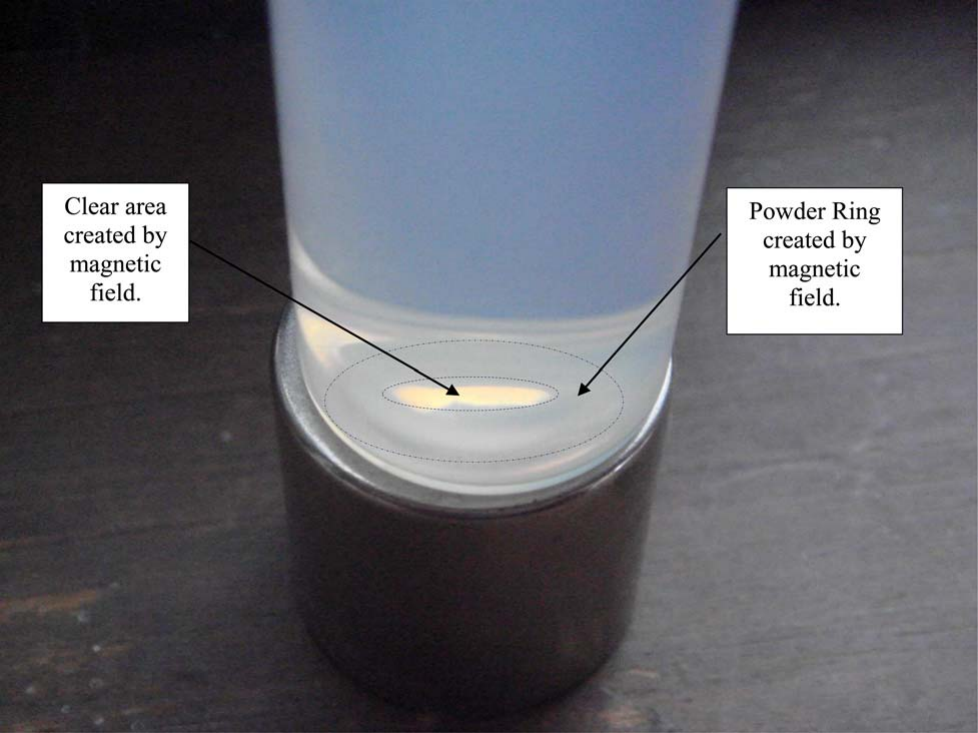
Base of the magnetic separation potash tube A with anatase titania in 0.01 N HCl exposed to ambient light. An outline of the powder ring indicates where the particles settled after being rejected by the magnetic field on day 30.

**Fig. 16:**
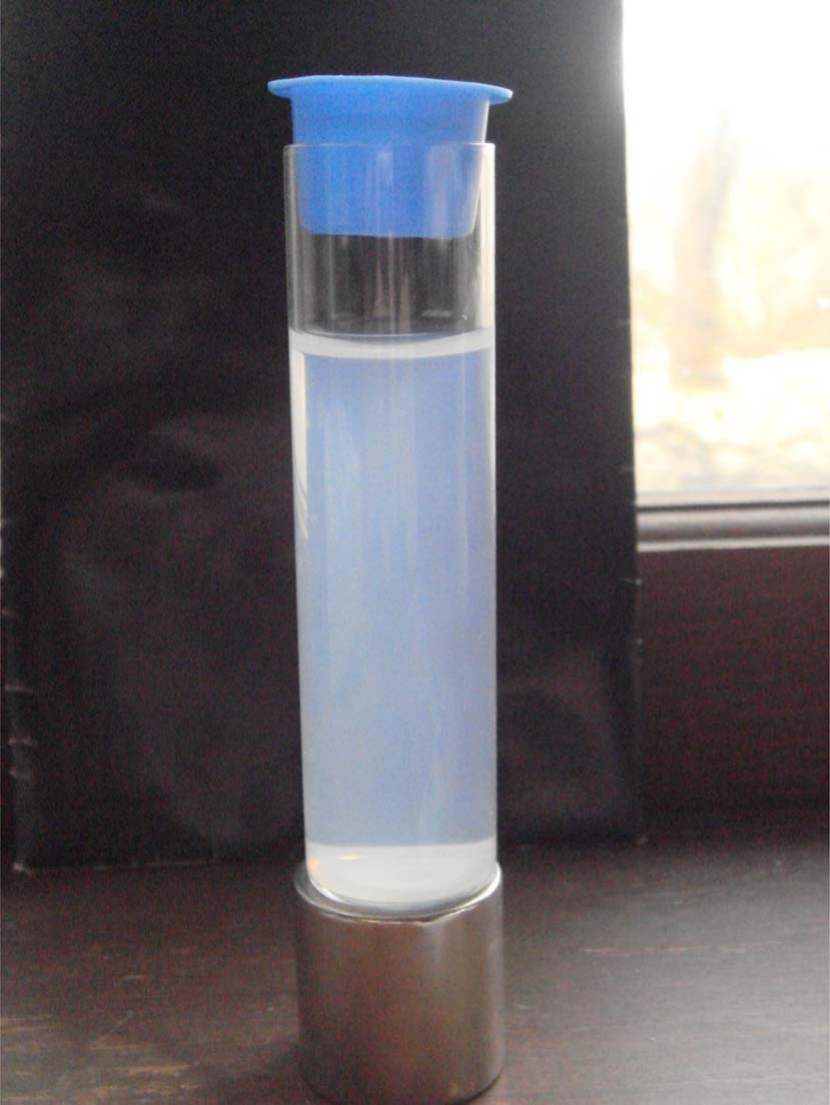
The final magnetic separation of anatase titania in 0.01 N HCl exposed to ambient light. The levitated particles are fully dispersed in the solution on day 43.

**Fig. 17:**
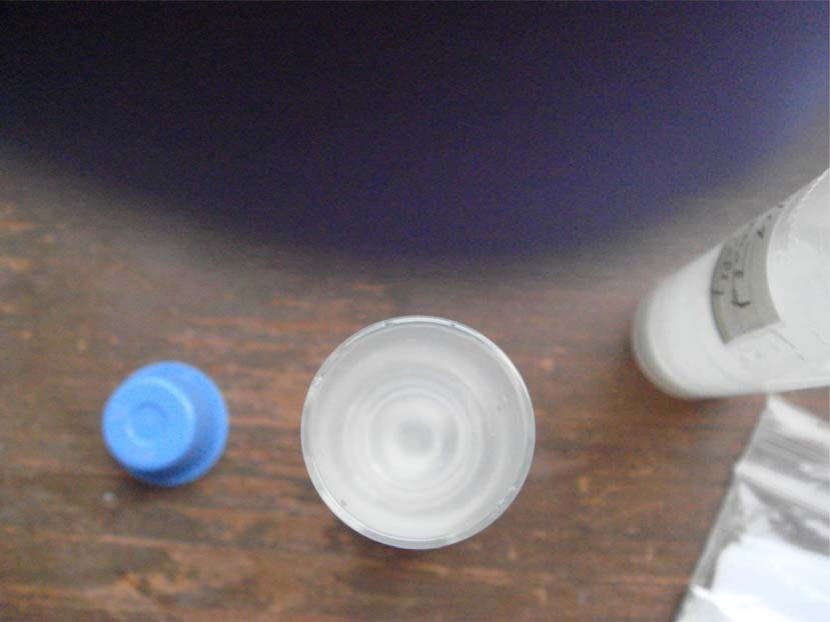
A view of the powder circles created by settling particles before the solution was removed on day 43. The concentric rings demonstrate how the settled particles were rejected by the strongest portion of the magnetic field during settling.

**Fig. 18:**
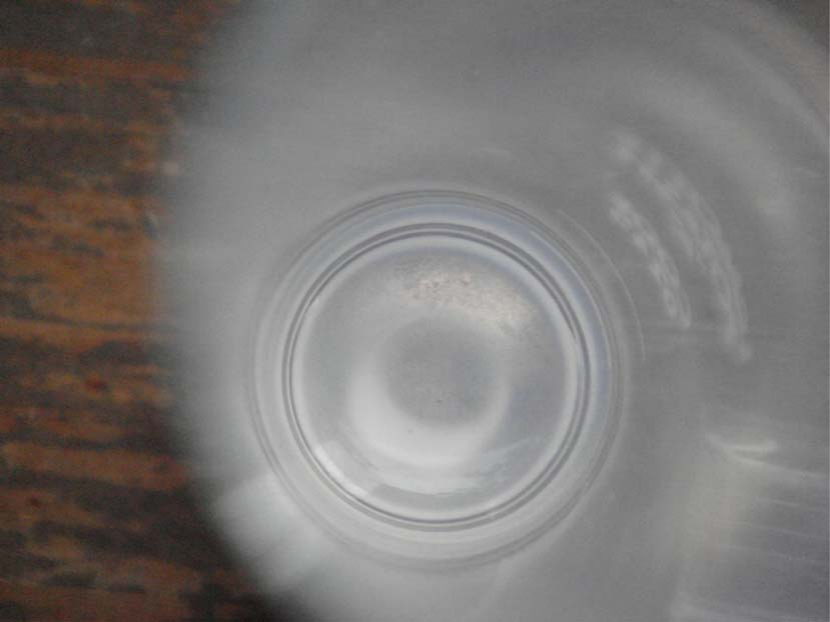
Magnetic separation of anatase titania dry powder residue after the removal of the 0.01 N HCl and evaporation of the remaining liquid on day 43. The powder at the base of the tube, with an empty circular region, is evidence that the particles were pushed out of the strongest part of the magnetic field as they settled.

### A.2 Settling of anatase titania particles in “Potash A” tube without the presence of a magnetic field

**Fig. 19:**
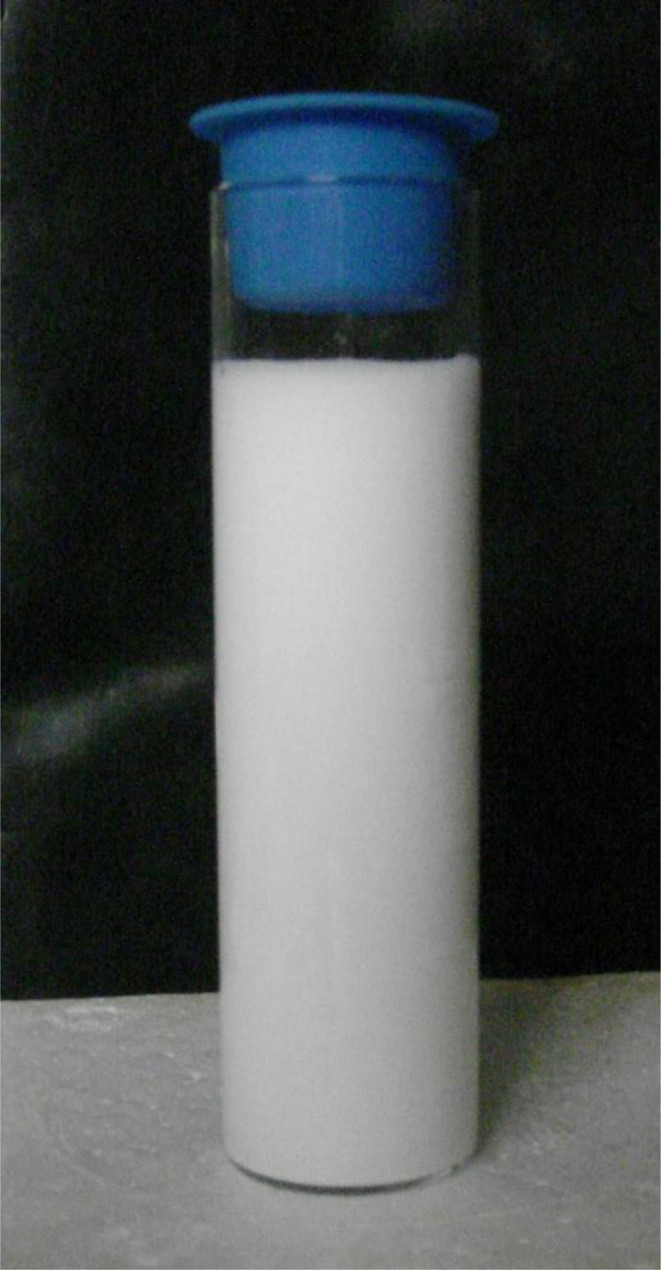
Disbursed anatase titania in deionized water for particle settling while not in the presence of a magnetic field.

**Fig. 20:**
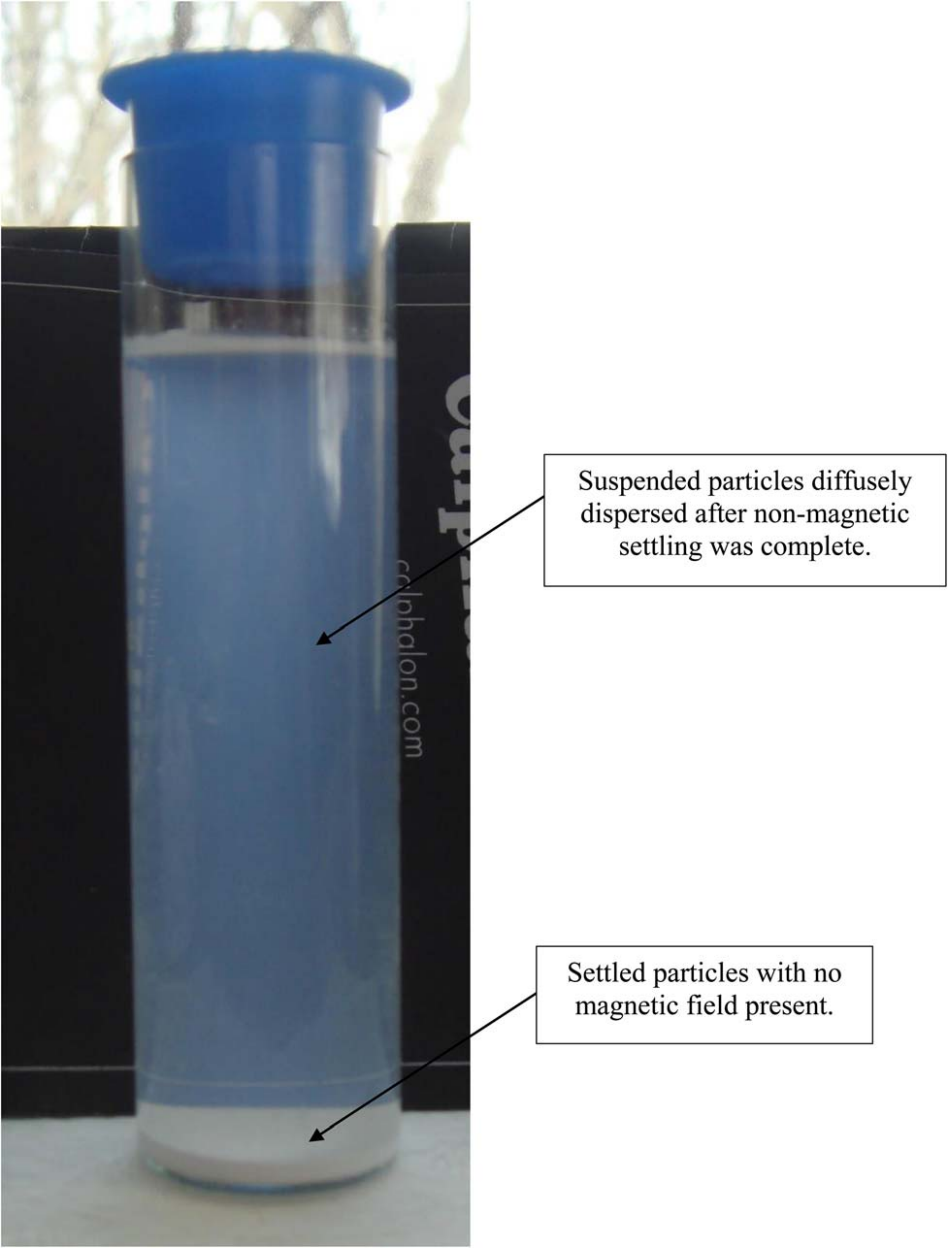
Anatase titania in deionized water while not in a magnetic field during settling while exposed to ambient light at 36 days.

**Fig. 21:**
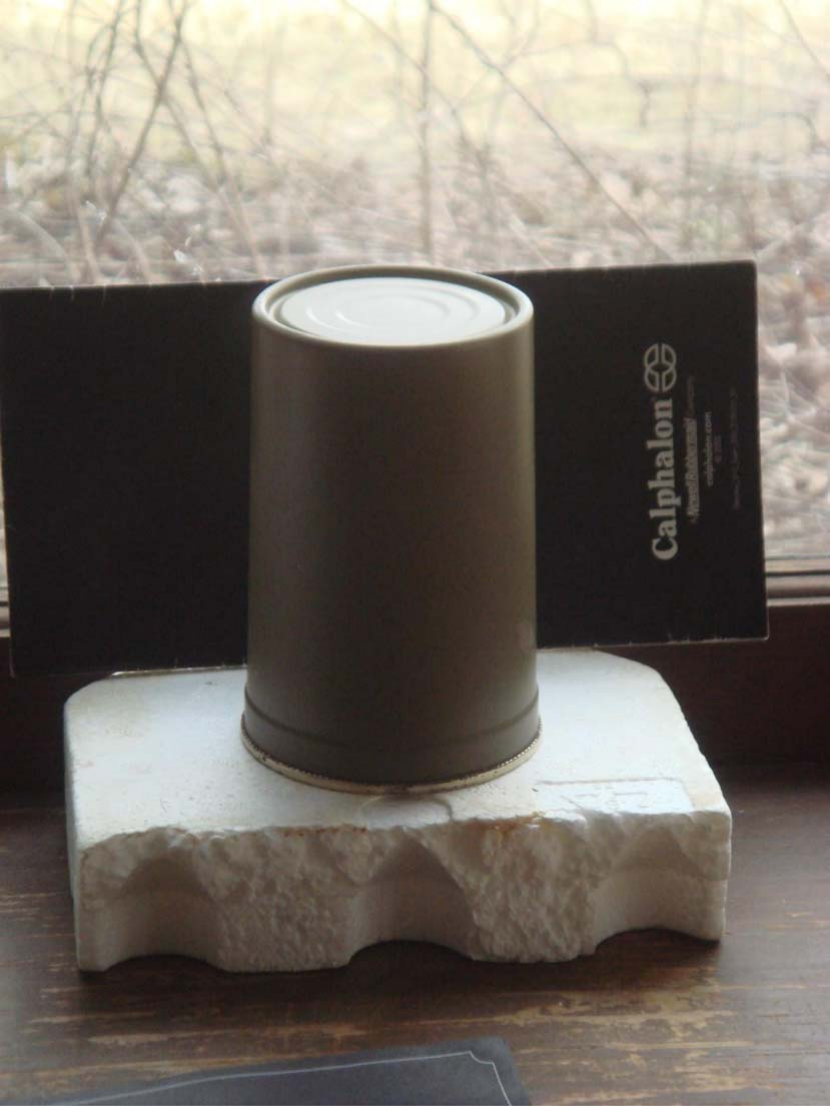
Metal cup set-up used to exclude ambient light during non-magnetic settling on day 53.

**Fig. 22:**
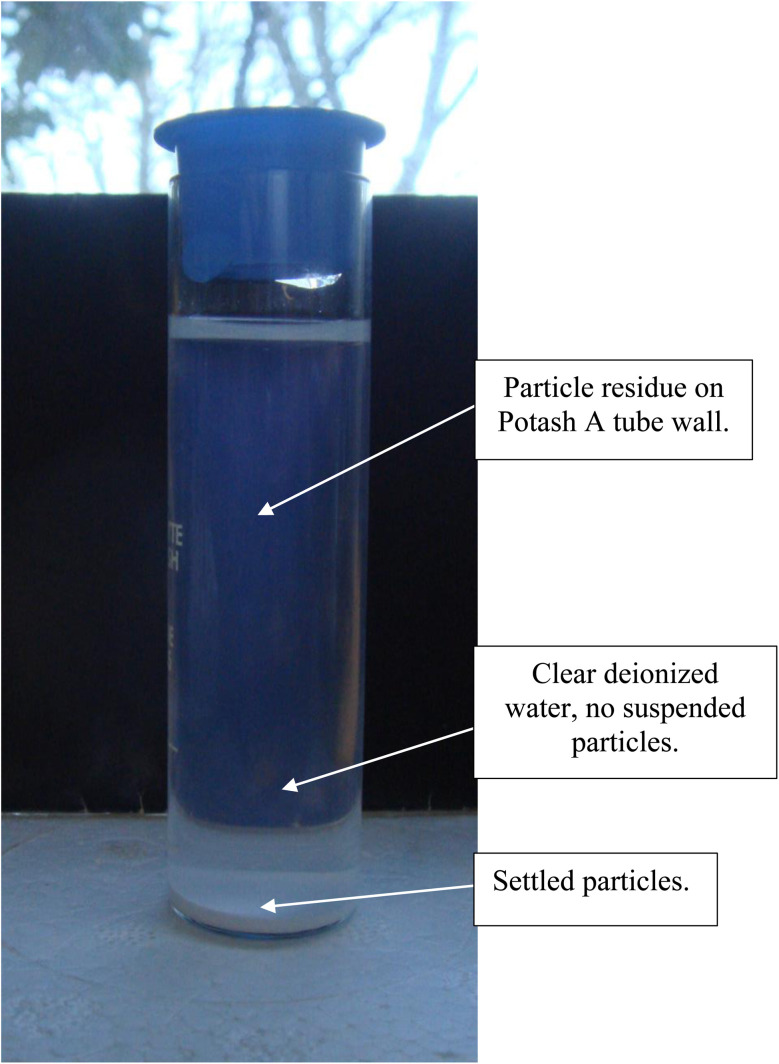
Complete settling of particles after ambient light was excluded on day 87.

### A.3 Magnetic separation of particles in deionized water with ambient light excluded

**Fig. 23:**
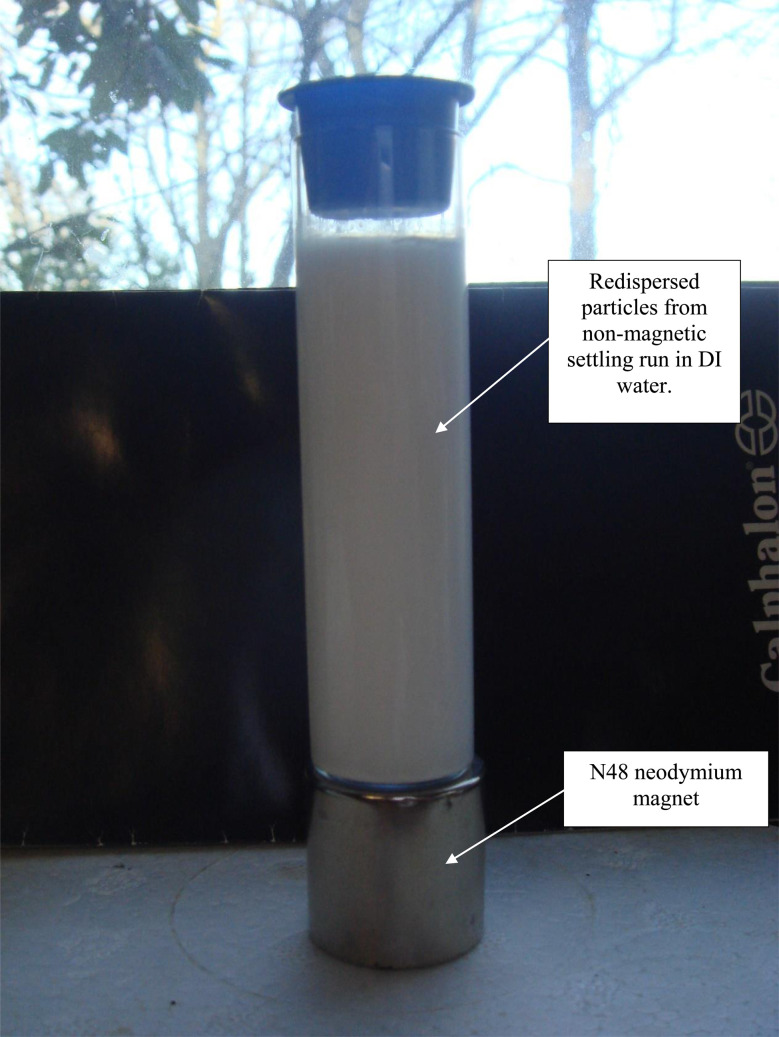
Redispersed particles from non-magnetic settling run.

**Fig. 24:**
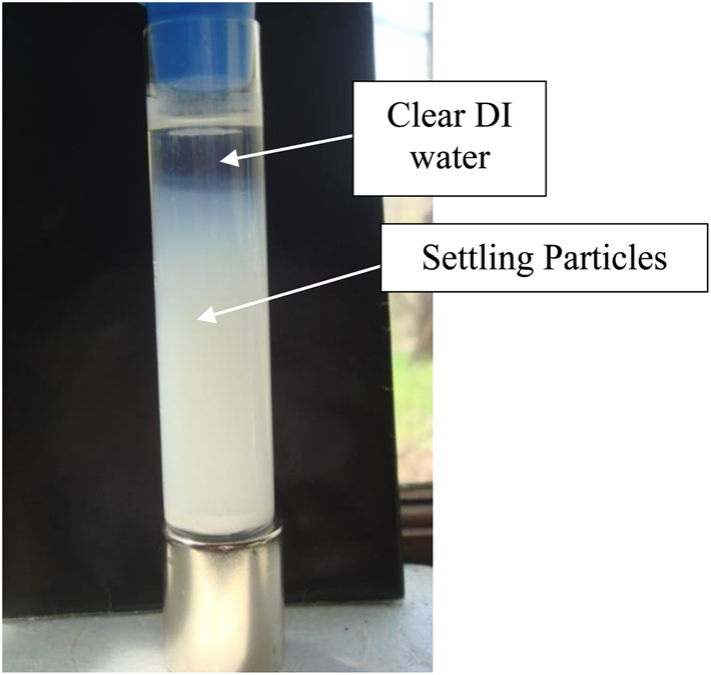
Magnetic separation on day 4.

**Fig. 25:**
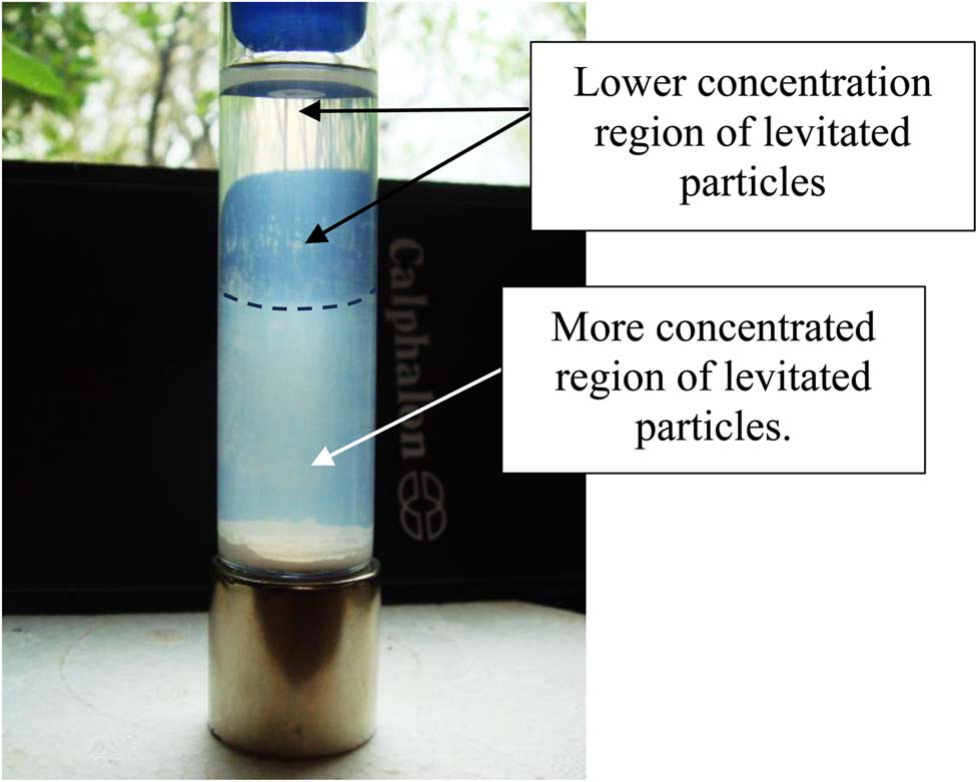
Magnetic separation on day 8. Above the dotted line the entire region is filled with a lower concentration of particles. Below the dotted line the suspended particle region is more concentrated. At the base of the tube are fully settled particles, unaffected by the magnetic field.

**Fig. 26:**
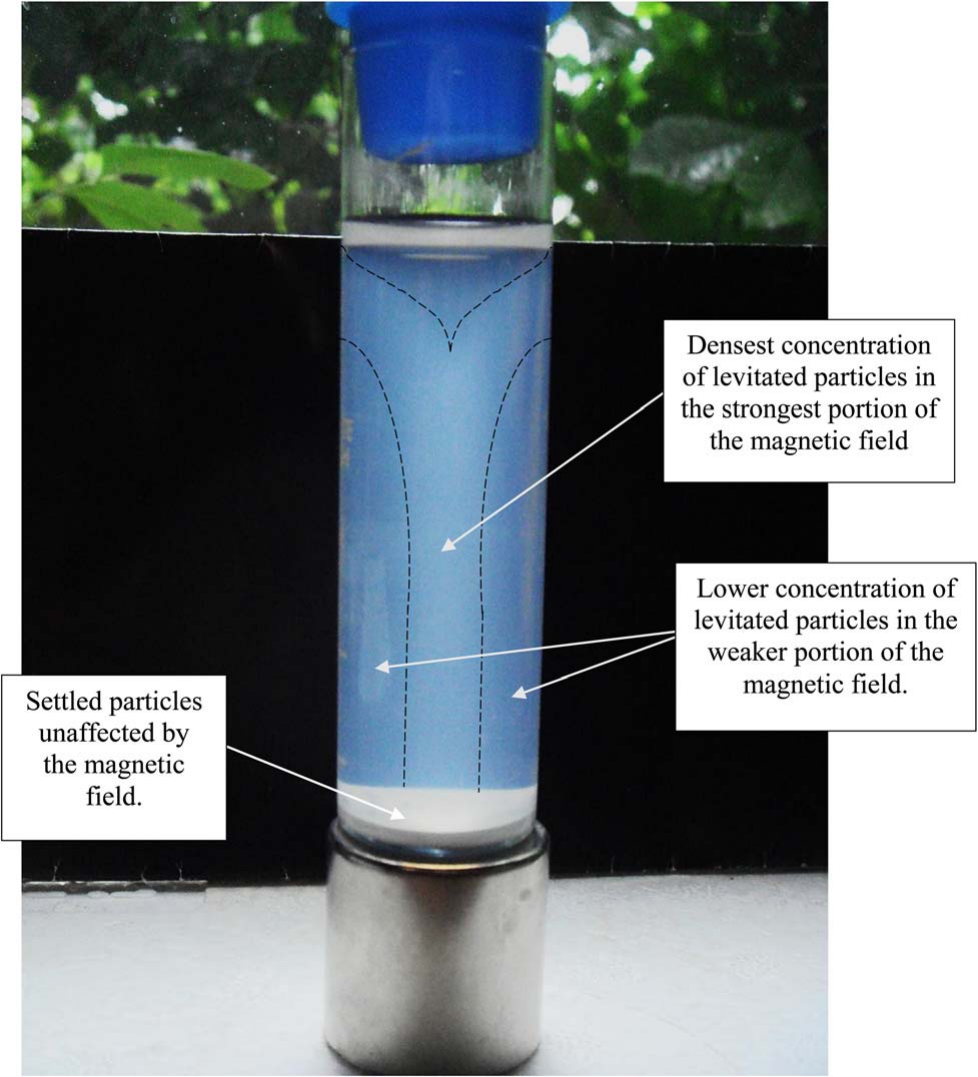
Magnetically separated anatase titania without ambient light at the end of the run, on day 15.

**Fig. 27:**
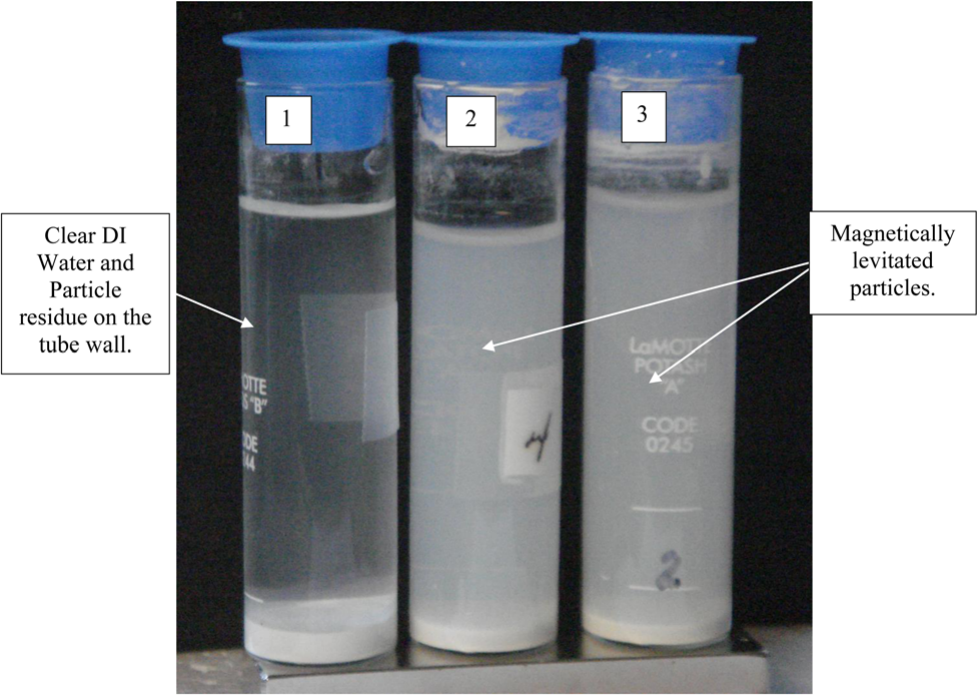
Completion of magnetically separated anatase titania without ambient light at the end of 15 days. Tube #1 exhibits only settled particles after 4 magnetic separation runs. These particles settled in approximately an hour after the beginning of the run. Tubes #2 and #3 still possess magnetically levitated particles as they have had fewer magnetic separation runs than Tube #1.

### A.4 Possible insights into the BCS theory

The findings in this work may offer possible insights into the BCS model^70^ for low and high temperature superconductors. These include the possible presence of a potential energy barrier at the exit/entrance of each conduction band at each particle's surface. The second is the possible way Cooper pairs interact with this barrier.

There is a strong possibility of a potential energy barrier at the surface of low and high temperature superconducting materials conduction band opening for particles above a critical particle size. In this work the critical average primary particle diameter was found to be *d* = 3.30 nm. This would create two interactions for an emerging Cooper pair. The first is a magnetic dipole–dipole interaction created by electron spin in the surface orbitals comprising the potential energy barrier. The second is an electrostatic interaction at the potential energy barrier, as would occur for two negatively charged electrons repelling each other.

The presence of Cooper pairs in low and high temperature superconductors would eliminate the magnetic dipole–dipole interactions. These paired electrons have spins of either 1 or 0 indicating they are perfectly matched^46^ making them perfectly diamagnetic.^47^ Electrons at 180° to each other cancel out each other's magnetic dipoles.^71,72^ Therefore, they would most likely not have interacted with the surface orbital magnetic dipole moments within the potential energy barrier created by the materials surface structure.

The second feature, electrostatic interaction of the Cooper pairs and the potential energy barrier may explain why high temperature superconductors exhibit a very small electrical resistance,^41–43^ even after the onset of superconductivity. If this electrostatic interaction does occur and is responsible for the very low electrical resistance below its *T*_C_ it suggests that the interaction is not a major factor in the overall electrical bulk resistance of the material. If these two interactions with a potential energy barrier do occur, then the magnetic dipole–dipole interaction is most likely responsible for most of the electrical bulk resistance in the low and high temperature superconductors above their *T*_C_.
